# Rgs1 is a regulator of effector gene expression during plant infection by the rice blast fungus *Magnaporthe oryzae*

**DOI:** 10.1073/pnas.2301358120

**Published:** 2023-03-13

**Authors:** Bozeng Tang, Xia Yan, Lauren S. Ryder, Mark Jave A. Bautista, Neftaly Cruz-Mireles, Darren M. Soanes, Camilla Molinari, Andrew J. Foster, Nicholas J. Talbot

**Affiliations:** ^a^The Sainsbury Laboratory, University of East Anglia, Norwich NR4 7UH, United Kingdom; ^b^School of Biosciences, University of Exeter, Exeter EX4 4QD, United Kingdom

**Keywords:** plant pathogen, rice blast, effectors, gene expression

## Abstract

To cause rice blast disease, the filamentous fungus *Magnaporthe oryzae* secretes a battery of effector proteins into host plant tissue to facilitate infection. Effector-encoding genes are expressed only during plant infection and show very low expression during other developmental stages. How effector gene expression is regulated in such a precise manner during invasive growth by *M. oryzae* is not known. Here, we report a forward-genetic screen to identify regulators of effector gene expression, based on the selection of mutants that show constitutive effector gene expression. Using this simple screen, we identify Rgs1, a regulator of G-protein signaling (RGS) protein that is necessary for appressorium development, as a novel transcriptional regulator of effector gene expression, which acts prior to plant infection. We show that an N-terminal domain of Rgs1, possessing transactivation activity, is required for effector gene regulation and acts in an RGS-independent manner. Rgs1 controls the expression of at least 60 temporally coregulated effector genes, preventing their transcription during the prepenetration stage of development prior to plant infection. A regulator of appressorium morphogenesis is therefore also required for the orchestration of pathogen gene expression required for invasive growth by *M. oryzae* during plant infection.

## Significance

Rice blast disease is a major threat to global food security. Successful plant infection by the blast fungus requires a battery of effector proteins to suppress plant immune responses, enabling the rapid invasion of plant tissue. How the blast fungus regulates expression of effector-encoding genes during plant infection is not known. We selected mutants that misregulated effector expression and identified a new regulator, Rgs1, previously implicated in control of appressorium development. Rgs1 represses effector gene transcription prior to the fungus infecting plant cells and derepresses their expression once invasive growth begins. The blast fungus therefore coordinates gene expression in a sophisticated and dynamic way to ensure that effectors are deployed at specific developmental stages during pathogenesis.

Plant pathogens secrete effector proteins into host tissues in order to suppress host immunity, modulate plant cell organization, and perturb cellular functions (1, 2). In this way, they reprogram host cells to facilitate pathogen invasion and proliferation. Effector gene expression is tightly regulated so that different families of effectors are deployed at each stage of plant infection. How plant pathogenic fungi regulate effector gene expression is, however, poorly understood. This is exemplified by the devastating rice blast pathogen *Magnaporthe oryzae* which possesses a large repertoire of effector genes expressed specifically during plant cell invasion (3). Although the function of effectors is an area of intense study (4), relatively little is known about the transcriptional control that governs effector gene expression (5–8).

In this study, we set out to investigate transcriptional regulation of effector-encoding genes in *M. oryzae* and how gene expression is orchestrated during plant infection. On the leaf surface, *M. oryzae* conidia germinate and sense plant surface cues which trigger the development of a specialized infection cell, the appressorium, required for penetration of host cells (9–11). Appressorium development requires heterotrimeric G-protein signaling, to transmit surface-sensing cues to downstream modules that facilitate morphogenesis, controlled by the mitogen-activated protein kinase Pmk1 (12, 13), and cAMP-dependent protein kinase A pathways (14). G-protein subunits in *M. oryzae* are controlled by regulator of G-protein signaling (RGS) proteins, which are important for appressorium development (15–18).

After penetration into rice tissue, the blast fungus rapidly switches to biotrophic growth and overcomes plant immunity by secreting effector proteins (19). At the tip of the primary invasive hypha, a plant-derived membrane-rich structure, the biotrophic interfacial complex (BIC), develops and remains intact as further secondary invasive hyphae fill the rice cell (20). Effectors of *M. oryzae* destined for delivery into plant cells, including avirulence gene products such as Avr-Pita, Avr-Pizt, Avr-Pii, and Pwl2, all localize to the BIC (21, 22), from which they appear to be translocated and delivered into host cells (5). A second group of effectors, including Slp1 and Bas4, are secreted to the apoplast where they suppress extracellular defence responses, such as chitin-triggered immunity (23). As a consequence of effector-mediated suppression of immunity, *M. oryzae* is able to proliferate rapidly in plant tissue and move from cell-to-cell using pit fields containing plasmodesmata. The fungus develops a specialized transpressorium at rice cell junctions, which facilitates cell invasion at pit fields in a process regulated by the Pmk1 MAPK pathway (24).

A recent transcriptional profiling study has shown that effector gene expression in *M. oryzae* occurs only during plant infection (25). Effector genes are temporally regulated during infection with early-acting effectors expressed as soon as 8 h after conidial germination, and large families of structurally conserved effectors, such as the Max effectors (26), expressed during biotrophic growth of the pathogen, 24 to 48 h after initial infection (25). The specific temporal and spatial expression patterns suggest that *M. oryzae* effectors must be under very precise transcriptional regulation, but little is known regarding the transcriptional regulators necessary to achieve this complexity of control. In other pathogenic fungi, our understanding of effector gene regulation is also limited. In the corn smut fungus *Ustilago maydis*, for instance, a transcriptional regulator Ros1 has been implicated in spore formation and effector gene expression during the late stages of infection (27), while in the necrotrophic wheat pathogen *Parastagonospora nodorum* the Zn_2_Cys_6_ transcription factor PnPf2 positively regulates 12 effector-like protein-encoding genes (28). Recent studies have also implicated global histone modification dynamics in control of pathogen gene expression in *M. oryzae* during infection (29). There are, however, only limited reports to date that have investigated the mechanism of effector gene regulation in plant pathogenic fungi (30).

In this study, we set out to identify putative regulators of effector gene expression. We reasoned that because effector genes are only expressed during growth in plant tissue, it would be possible to select for mutants that exhibit constitutive effector gene expression. These would potentially carry mutations in genes encoding transcriptional regulators. Here, we report a simple forward genetic screen using a strain of *M. oryzae* in which we expressed a translational fusion of an effector, Mep2, with a green fluorescent protein tag. Using this reporter line, we selected *M. oryzae* mutants in which we could observe constitutive Mep2-GFP fluorescence in hyphae and spores. This led to the identification of a mutant, *cer7*, which carries a single-point mutation in a gene called *RGS1*. We show that the Rgs1 protein—which has been previously implicated as a regulator of G-protein signaling during appressorium development by *M. oryzae* (15–17)—also acts as a transcriptional regulator of effector gene expression. We provide evidence that Rgs1 is necessary for repressing the expression of at least 60 temporally coregulated effector-encoding genes during the prepenetration stages of development and that these genes are subsequently derepressed during invasive growth by the fungus enabling their specific deployment in plant tissue.

## Results

### A Forward Genetic Screen to Identify Regulators of Effector Gene Expression in *M. oryzae*.

**Fig. 1.**
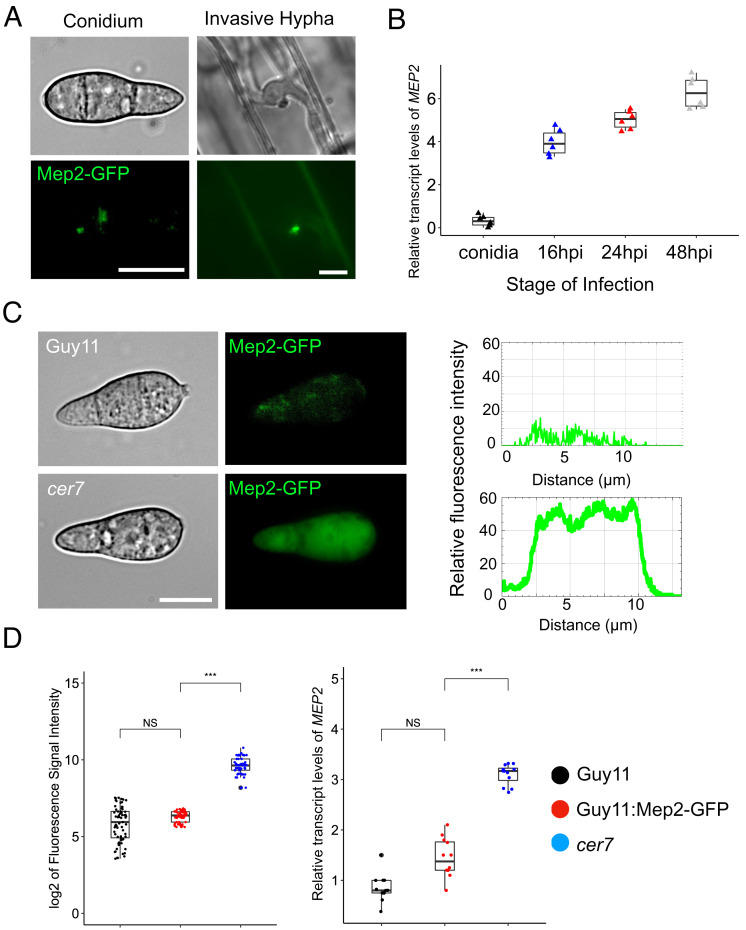
A forward genetic screen identified the *cer7* mutant of *M. oryzae* (*A*) Micrographs showing differential expression of Mep2-GFP at the BIC in invasive hyphae and basal expression in conidia of the wild-type *M. oryzae strain* Guy11. (Scale bar, 10 µm.) BIC localization was imaged in rice leaf sheath tissue inoculated with conidia of Guy11:Mep2-GFP at 32 hpi. (*B*) Boxplots to show relative transcripts of *MEP2* as log2 fold changes values in invasive hyphae of Guy11. The samples were harvested from Guy11. conidia and leaf sheath harvested at 16 hpi, 24 hpi, and 48 hpi. n = 6 experiments. Expression is shown relative to the *M. oryzae* actin gene. (*C*) Micrographs and line-scan graphs showing the constitutive fluorescence signal of Mep2-GFP in mutant strain *cer7* conidia, compared to the wild-type Guy11 (Scale bar, 10 µm.) (*D*) Box plots to show fluorescence intensities of Mep2-GFP as log2 values and relative abundance of *MEP2* transcripts as log2 fold change values in qRT-PCR. Colours correspond to Guy11 (black), *cer7* (red), and Guy11:*MEP2-GFP* (blue). Significance between groups of samples was performed using Unpaired Student’s *t* test. ***P < 0.001, *P < 0.05, NS = no significant difference.

In this study, we set out to identify transcriptional regulators of effector gene expression in *M. oryzae*. We reasoned that because effectors are only expressed during plant infection, selecting mutants that show constitutive expression of an effector gene would provide a simple method to identify corresponding regulatory gene, carrying either a mutation leading to constitutive activation of a transcriptional activator, or a loss of function mutation in a repressor, for example. We therefore generated a strain of *M. oryzae* expressing an effector-encoding gene *MEP2*, fused to the green fluorescent protein gene (GFP). *MEP2* was identified in a recent study that characterized the effector repertoire of *M. oryzae* based on their differential expression during pathogenesis (25). *MEP2* shows peak expression 48 h after infection based on RNA-seq analysis, corresponding to a time of rapid plant tissue biotrophic colonization by the fungus (25). We generated a *MEP2:GFP* gene fusion, transformed it into a *M. oryzae* wild-type rice pathogenic strain Guy11 and selected a transformant with a single integration of the reporter gene construct. We observed specific expression and localization of Mep2-GFP fluorescence in the biotrophic interfacial complex (BIC) of invasive hyphae during rice infection, with very little detectable expression in either conidia or vegetative hyphae of the fungus (Fig. 1*A* and *SI Appendix*, Fig. S1*B*). Consistent with this, quantitative real-time PCR (qPCR) showed high transcript abundance of *MEP2* in invasive hyphae (IH) with peak expression at 48 h postinoculation (hpi) compared to minimal basal expression in conidia (Fig. 1*B*). Having established that Mep2-GFP is specifically expressed during in planta growth by *M. oryzae*, we carried out UV mutagenesis on conidia of the Mep2-GFP strain. We selected mutants that showed constitutive Mep2-GFP fluorescence in conidia and named them Cer (Constitutive Effector Regulator) mutants. One of these mutants, *cer7,* which exhibits the highest signal of green fluorescence in conidia, was selected (Fig. 1*C* and *SI Appendix*, Fig. S1*A*). We verified constitutive expression of *MEP2* using qPCR which showed elevated expression in *cer7*, compared to the original Mep2-GFP transformant (Fig. 1*D*). We also observed constitutive expression of Mep2-GFP in mycelium grown in axenic culture, in appressoria, and in the BIC of invasive hyphae in the *cer7* mutant (*SI Appendix*, Fig. S1*B*). Taken together, these results suggest that the expression of the *MEP2* effector gene is induced, or derepressed in spores and mycelium of the *cer7* mutant.

### Identification of the *CER7* Locus by Bulked Segregant Analysis.

**Fig. 2.**
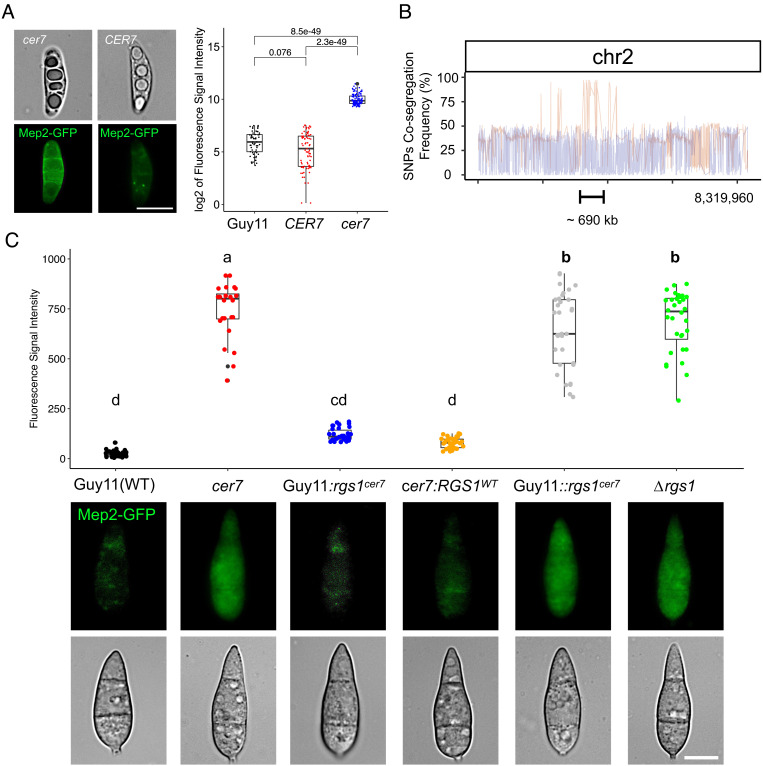
Bulked segregant analysis and genetic complementation defined *rgs1^cer7^* as the allele responsible for constitutive expression of Mep2-GFP in conidia. (*A*) Micrographs showing expression of Mep2-GFP in segregating ascospores obtained from a cross between *cer7* and TH3. A single ascospore was isolated from an ascus under the dissecting microscope. Progeny displaying a high level of fluorescence signal from Mep2-GFP (*cer7*) were pooled together for whole genome sequencing. (Scale bar, 2 µm.) Boxplots showing log2 values of fluorescence intensities of Mep2-GFP measured from conidia of 253 individual *M. oryzae* progeny. Progeny with *cer7* phenotype (blue), and those showing *CER7* phenotype (red), were compared to wild-type Guy11 conidia (black). n(*cer7*) = 59, n(*CER7*) = 194. A t test was performed to determine the significance between the samples and *P* values are shown. (*B*) Graph showing SNP cosegregation frequencies on chromosome 2 after sequencing genomic DNA from pools of progeny segregating for *cer7* and *CER7* phenotypes, respectively. Red line shows frequencies of variants identified from pooled genomic DNA from progeny showing the *cer7* phenotype. Blue line shows frequencies of variants identified from pooled genomic DNA from progeny with *CER7* phenotype (*C*) Boxplot and micrographs to show the fluorescence intensity of Mep2-GFP in conidia of wild-type Guy11 (Black), *cer7* (red), Guy11:*rgs1^cer7^* (blue), *cer7:RGS1^WT^* (orange), Guy11::*rgs1^cer7^* (gray), and Δ*rgs1* (green). Letters within each sample refer to one-way ANOVA tests (*P* < 0.05, Duncan test). Micrographs show bright field and epifluorescence images of conidia. (Scale bar, 10 µm.)

To identify the mutation leading to constitutive *MEP2* expression in the *cer7* mutant, we first sequenced the genome of *cer7* mutant and aligned it against the genome sequence of the original Guy11 Mep2-GFP transformant and the *M. oryzae* reference genome of strain 70-15 (31). A total of 1,955 variants (SNPs and indels) were identified compared to the *M. oryzae* 70-15 reference genome sequence of which 1,036 were located within the coding regions of 408 different genes. To identify the *cer7* mutation, we carried out bulked segregant analysis (32) by crossing the *cer7* (*Mat1*-*1*) mutant with a wild-type strain TH3 of opposite mating type (*Mat1*-*2*) (*SI Appendix*, Fig. S2*A*). We selected perithecia and dissected asci (*SI Appendix*, Fig. S2*B*). Ascospore progeny were then collected and phenotypically characterized based on the fluorescence signal of Mep2-GFP (Fig. 2*A*). A total of 253 progeny were selected, of which 59 progeny (23.3%) showed the *cer7* phenotype, and 194 progeny the wild-type phenotype (Fig. 2*A*). The *MEP2-GFP* construct is present in a single copy in the *cer7* mutant, as confirmed by de novo assembly of the *cer7* genome sequence and would therefore be predicted to segregate in a 1:1 ratio in ascospore progeny. We reasoned that if *cer7* is caused by mutation at a single locus then this should also segregate in a 1:1 ratio. We would therefore expect to see the observable *cer7* fluorescent conidia phenotype in a 1:3 ratio, with a quarter of progeny showing constitutive Mep2-GFP expression, which was validated by a Chi-squared test (χ^2^ = 0.135, df = 1, *P* = 0.713). To carry out bulked segregant analysis, we then extracted genomic DNA from *cer7* and wild-type progeny, respectively, and bulked them into two separate pools for genome sequencing. This enabled us to define a region of 692 kb on supercontig 8.2 which showed the highest frequency of SNPs identified in progeny showing the *cer7* phenotype (Fig. 2*B*). Within this region, only one polymorphism matched SNPs identified in the genome sequence of the *cer7* mutant, located at position 3779156 in the coding region of gene MGG_14517. This gene has previously been identified as *RGS1*, which encodes a regulator of G-protein signaling in *M. oryzae* (15). The SNP results in a single amino acid sequence change in the predicted gene product from glutamic acid to a stop codon (GAA to TAA) (*SI Appendix*, Fig. S2*C*). To confirm the association, we sequenced PCR-amplified fragments spanning the SNP in 10 *cer7* and 10 wild-type progeny (*SI Appendix*, Fig. S2*D*), which verified the analysis.

To test whether the *RGS1* gene corresponds to *cer7*, we carried out genetic complementation and allelic replacement assays. We first introduced the wild-type *RGS1^WT^* allele into the *cer7* mutant, which resulted in transformants with nonfluorescent conidia (Fig. 2*C*). Introducing the *rgs1^cer7^* allele ectopically into the wild-type (*RGS1^+^*) Mep2-GFP strain also resulted in nonfluorescent conidia. By contrast, when we carried out targeted allelic replacement of *RGS1* with the *rgs1^cer7^* mutant allele in the Mep2-GFP strain, this resulted in transformants with fluorescent conidia. When considered together, this provides evidence that *cer7* is a recessive loss of function mutation in the *RGS1* gene, consistent with the premature stop codon generated by the mutation (Fig. 2*C*). To test this idea directly, we generated a targeted gene deletion mutant in the wild-type Mep2-GFP background. This led to constitutive expression of Mep2-GFP in conidia of the resulting Δ*rgs1* mutants (Fig. 2*C*). The results were validated by qPCR which confirmed that *cer7* and Δ*rgs1* mutants show high level expression of *MEP2* in conidia (*SI Appendix*, Fig. S3*A*). We also observed the Δ*rgs1* and *cer7* strains produced similar phenotypes in axenic culture, showing white aerial hyphal growth, water soaking, and aberrant appressorium formation on hydrophilic surfaces, which is consistent with previous reports of Δ*rgs1* mutants (15, 16, 33) (*SI Appendix*, Fig. S3*B*).

### Rgs1 Acts as a Transcriptional Regulator of the *MEP2* Effector Gene.

**Fig. 3.**
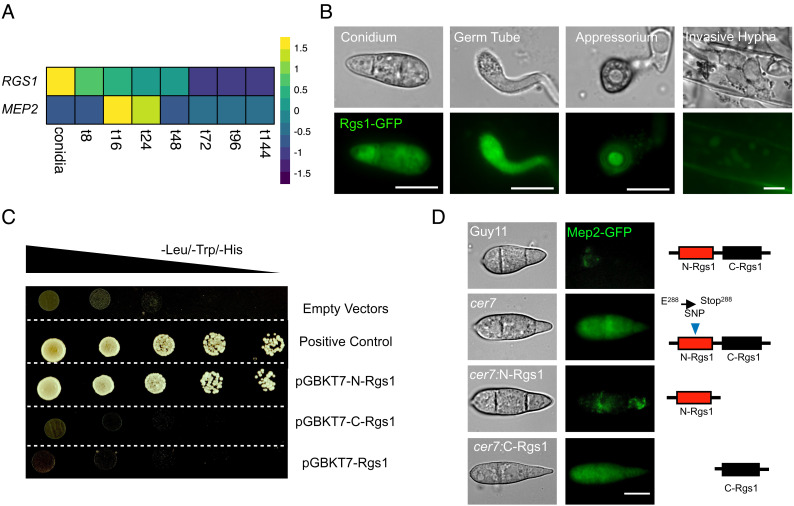
The N terminus of Rgs1 is required for the repression of *MEP2* expression in conidia. (*A*) Heatmap showing relative transcript abundance of *MEP2* (MGG_00230) and *RGS1* (MGG_14517) genes in *M. oryzae* conidia, and during infection from 8 to 144 hpi. Relative transcript levels are fold change compared to expression in mycelium (from data set PRJEB44745). Data were extracted from RNA-seq dataset PRJEB45007. The colour key shows scaled fold change values. (*B*) Micrograph showing the fluorescence signal of Rgs1-GFP expressed in Guy11. Live-cell imaging was performed during a time course experiment to investigate expression of Rgs1-GFP in conidia, germ tubes, mature appressoria, and invasive hyphae at 24 hpi. (*C*) Images showing transactivation activity of N-terminal Rgs1 (N-Rgs1), C-terminal Rgs1 (C-Rgs1), and full length Rgs1 in yeast cells. Cotransformation of Y2HGold yeast cells with bait (BD) and prey (AD) vectors was carried out with following combinations; pGBKT7-N-Rgs1/pGADT7, pGBKT7-C-Rgs1/pGADT7, pGBKT7-Rgs1/ pGADT7, and positive control (pGBKT7-53 and pGADT7-T) along with empty vectors, and grown in double drop-out and quadruple-dropout media. Images represent two independent biological replicates. (*D*) Micrographs showing expression of Mep2-GFP in conidia of strains *cer7*, Guy11, *cer7*:N-Rgs1, *cer7*:C-Rgs1. Conidia from each strain were harvested from colonies after 5 d growth on CM and immediately mounted on microscope slides for GFP visualization using epifluorescence microscopy. (Scale bar, 10 µm.) The schematic illustration demonstrates different genotypes in corresponding *M. oryzae* strains.

Rgs1 has been studied previously in *M. oryzae* as a regulator of G-protein signaling which affects asexual development, appressorium formation, surface sensing, and virulence through its interaction with the three Gα subunit proteins MagA, MagB, and MagC (15). Mutants lacking Rgs1 form appressoria on noninductive hydrophilic surfaces and show reduced virulence resulting from misregulation of MagA, as well as a hypersporulation phenotype associated with misregulation of MagB (33). The reported roles of Rgs1 are therefore associated with the prepenetration phase of development, prior to plant tissue invasion (16, 17, 33). To investigate the potential role of Rgs1 as an effector regulator, we therefore investigated the temporal expression of *RGS1* in publicly available RNA-seq dataset (PRJEB45007) (25). This showed that *RGS1* is expressed in conidia and during the initial stages of appressorium formation, but then very reduced in expression during plant infection. This is the reciprocal pattern to *MEP2*, which is not expressed in conidia, but highly expressed during invasive growth, peaking in expression at 48 h after infection (Fig. 3*A*). To experimentally verify this pattern of expression, we generated a *M. oryzae* transformant expressing Rgs1-GFP and carried out live cell imaging. Rgs1-GFP is highly expressed in conidia, germ tubes, and incipient appressoria, but significantly reduced in invasive hyphae (Fig. 3*B*). We then extracted total protein from mycelium and plant tissue infected with the Rgs1-GFP strain, and a control strain of *M. oryzae* expressing GFP under control of a high-level constitutive promoter ToxA (34), and performed western blot analysis with anti-GFP antibodies. We detected the predicted 106 kDa Rgs1-GFP fusion protein in mycelium, but this was not observed in infected plant tissue samples at 32 h after infection (*SI Appendix*, Fig. S4*A*). To investigate the nature of Rgs1 expression further, we next analyzed published RNA-seq data (PRJEB36580) (35) and found high coverage reads spanning all three exons of *RGS1* from conidia, while aligned reads from the first exon of *RGS1* were very reduced in RNA-seq data from infected plant material at 24 hpi (*SI Appendix*, Fig. S4*B*). This suggests that exon skipping of *RGS1* may occur during plant infection, resulting in a lower abundance of the N-terminal domain of the Rgs1 protein. It has previously been reported that Rgs1 undergoes endoproteolytic cleavage which leads to the generation of a tandem Dishevelled, Egl-10, Pleckstrin domain (DEP–DEP) protein from the N terminus of Rgs1 (N-Rgs1), encoded by the first and second exon, and a separate RGS core domain protein from the C terminus (C-Rgs1) (33). It has been proposed that the N-Rgs1 protein is required for vesicular membrane targeting of the protein, while the C-Rgs1 protein is sequestered in the vacuole, providing a post-translational mechanism to regulate the catalytic activity of Rgs1 on its Gα subunit substrates. Given the low level of N-Rgs1—associated transcripts during plant infection, we wondered whether the effector regulation function resided in N-Rgs1 and occurred during the prepenetration phases of development. To investigate whether N-Rgs1 can act as a transcription factor, we therefore tested its transactivation activity and DNA-binding ability in yeast. We found that when N-Rgs1 is fused to the Gal4 DNA-binding domain it is able to act as a transcriptional activator (Fig. 3*C*), but when fused to the Gal4 activation domain, it is unable to bind DNA (*SI Appendix*, Fig. S5*A*). Meanwhile, neither the full length Rgs1 nor the C-Rgs1 protein show any transactivation or DNA-binding activity (*SI Appendix*, Fig. S5*A*). These results suggest that N-Rgs1 might function independently to regulate *MEP2* transcription, consistent with the position of the *cer7* premature stop codon mutation and the potential that exon skipping of Rgs1 takes place during plant infection. To test this idea, we constructed vectors carrying sequences encoding N-Rgs1 or C-Rgs1, respectively, driven by the native *RGS1* promoter and terminator sequences, and transformed these into the *M. oryzae cer7* mutant. We found that N-Rgs1 was able to complement *cer7* preventing expression of Mep2-GFP in conidia (Fig. 3*D*). By contrast, expressing C-Rgs1 did not complement the *cer7* phenotype and conidia remained fluorescent (Fig. 3*D*). In control experiments, we verified *cer7* complementation with the full-length *RGS1* gene and lack of complementation with the *RGS1^cer7^* allele. When considered together, these results suggest that N-Rgs1 acts as a repressor of transcription of *MEP2* in conidia, preventing its expression prior to plant infection. However, given that N-Rgs1 is unable to bind DNA, which was also confirmed using a yeast-one hybrid assay which did not find any evidence for N-Rgs1 binding to the *MEP2* promoter (*SI Appendix*, Fig. S5*B*), it is likely that N-Rgs1 regulates transcription of *MEP2* indirectly, perhaps by activating a repressor protein, or acting in association with another partner to bring about *MEP2* repression. We conclude that N-Rgs1 is necessary for regulation of the *MEP2* effector gene.

### Rgs1 Regulates Effector Gene Expression Independently of its G-protein Signaling Function.

Rgs1 has been reported to accelerate the intrinsic GTPase activity of target Gα subunits during appressorium development (15), and we therefore decided to test directly whether its RGS activity, which resides in the C-Rgs1 part of the protein, was associated with its ability to regulate *MEP2* transcription. We decided to test whether repression of *MEP2* transcription during the prepenetration stage of development is regulated via the action of Rgs1 on its associated Gα subunits in response to surface cues, thereby enabling Gα subunits to activate their downstream effector modules to trigger *MEP2* transcription. We reasoned that if *MEP2* transcription is G-protein-dependent, then mutants affected in their sensitivity to Rgs1 signaling, lacking GTPase activity, or constitutively activated GTP-Gα-subunits, would be affected in *MEP2* transcription. To test this idea, we generated Rgs1-insensitive alleles MagA(G187S), MagB(G183S), and MagC(G184S), the GTPase-inactive MagA(Q208L) and MagB(Q204L) alleles, and the MagB(G42R) constitutively activated allele (15), and introduced them each into the Guy11 strain expressing Mep2-GFP. We did not observe increased Mep2-FP expression in conidia of any of these transformants (*SI Appendix,* Fig. S5*C*). We also generated *ΔmagA*, *ΔmagB*, and *ΔmagC* targeted deletion mutants in the *cer7* mutant background, respectively, and all of the mutants showed constitutive expression of Mep2-GFP (*SI Appendix,* Fig. S5*D*). Taken together, these results provide evidence that the N-Rgs1 protein is necessary to regulate transcription of *MEP2* in conidia and acts independently of the G-protein signaling function of Rgs1.

### Rgs1 Regulates a Large Group of Effector Genes in *M. oryzae*.

**Fig. 4.**
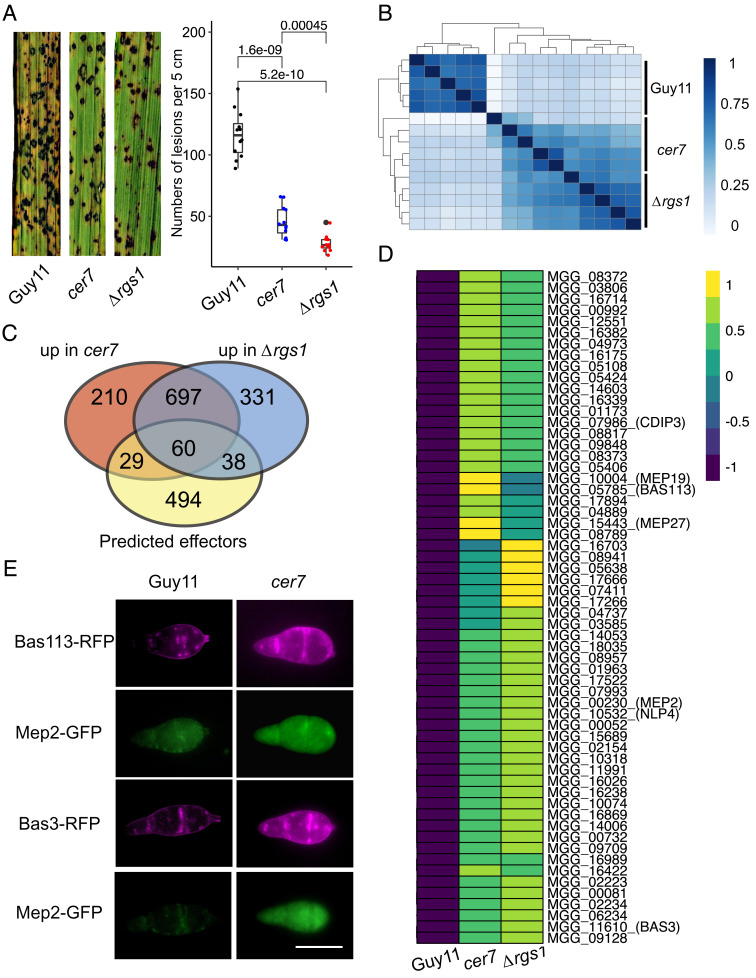
Rgs1 Regulates expression of a subpopulation of effectors during plant infection. (*A*) Seedlings of rice cultivar CO-39 were inoculated with *M. oryzae* conidial suspensions of equal concentration (1 × 10^5^ conidia/mL) of wild-type Guy11, *cer7* and Δ*rgs1* mutants. The boxplot represents the number of rice blast disease lesions per 5 cm in three independent repetitions of the experiment. Unpaired Student’s *t* test was performed to determine significant differences. (*B*) Heatmap showing the Euclidean distance between RNA-seq samples from conidia of *cer7*, the wild-type Guy11, and Δ*rgs1*. Normalized reads counts were used from all the samples to determine clustering. Intensity of colours represent similarities and distance between samples. (*C*) Venn diagram to show the number of effector genes derepressed in conidia of the *cer7* and Δ*rgs1* mutants. Blue circle = *cer7*, red circle = Δ*rgs1*. (D) Heatmap showing expression of 60 effector genes significantly upregulated in conidia of *cer7* and Δ*rgs1* mutants, compared to Guy11. Normalized expression values of transcripts used the TMM method. (*E*) Micrographs showing expression of Mep2-GFP, Bas113-RFP, and Bas3-RFP in conidia of *cer7* compared to Guy11. Conidial suspensions from each strain were inoculated onto hydrophobic glass coverslips and imaged using epifluorescence microscopy. (Scale bar, 10 µm.)

To determine the wider function of Rgs1, we tested the ability of the Δ*rgs1* and *cer7* mutants to cause rice blast disease. We inoculated 21-d-old seedlings of the susceptible rice cultivar CO-39 and scored disease symptoms after 5 d. A significant reduction in disease lesion number was observed in Δ*rgs1* and *cer7* infections compared to the isogenic wild-type Guy11 (Fig. 4*A*). Rgs1 has previously been reported to play a role in pathogenesis, but this has been attributed to its G-protein regulatory function (11). We decided to test whether Rgs1 regulates a wider group of virulence-associated genes by performing comparative global transcriptional profiling using Δ*rgs1* and *cer7* mutants compared to Guy11. We reasoned that genes regulated by Rgs1 during plant infection would show a similar derepression in conidia of Δ*rgs1* and *cer7* mutants. We therefore extracted mRNA from conidia Δ*rgs1*, *cer7*, and Guy11 using a total of five biological replicates of the experiment and performed RNA-seq analysis. Euclidean analysis was used to determine the similarity between expression profiles in each mutant, revealing a strong overlap between Δ*rgs1* and *cer7* mutants (Fig. 4*B*). This was consistent with principal component analysis which also demonstrated very similar transcriptional patterns between the *cer7* and Δ*rgs1* mutant (*SI Appendix*, Fig. S6*A*). In total, 996 genes were upregulated in conidia of *cer7*, and 1126 in Δ*rgs1* compared to Guy11 (log2|FC|>1, padj<0.05). Of these, 757 are shared between *cer7* and Δ*rgs1*. Metabolic enrichment analysis of the Rgs1-repressed gene set showed over-representation of gene functions associated with starch and sucrose metabolism, and glycan degradation, which are also induced during biotrophic invasive fungal growth, as well as phenylalanine and tyrosine metabolism which may be associated with secondary metabolism (*SI Appendix*, Fig. S6*B*). We also observed over-representation of biological processes associated with membrane function and transmembrane transport, as well as oxido-reductases. These functions too are associated with biotrophic invasive growth of *M. oryzae* (25) (*SI Appendix*, Fig. S6*C*). A recent study of the transcriptional landscape of plant infection by *M. oryzae* used weighted gene co-expression network analysis (WCGNA) to define temporal coexpression clusters of *M. oryzae* genes during infection (25) (*SI Appendix*, Fig. S7*A*). We observed that Rgs1-regulated genes can be classified into each WCGNA cluster but genes upregulated in conidia of Δ*rgs1* mutants are enriched in cluster M5, which peaks in expression at 48 h after infection (*SI Appendix*, Fig. S7*A*). This cluster also contains many effector-encoding genes (25). Consistent with this, we found 98 predicted or known effector-encoding genes upregulated in conidia of Δ*rgs1* mutants, of which 60 were also upregulated in conidia of the *cer7* mutant (Fig. 4*C*) (*SI Appendix*, Table S3). This suggests that in addition to *MEP2*, Rgs1 may regulate a much larger group of effectors, that are derepressed in conidia when the function of Rgs1 is compromised. Among this group, there were eight previously reported effector candidates, including *BAS3*, *BAS113*, *MEP19*, and *MEP27* (8, 25), and the necrosis and ethylene-inducing (Nep-like) peptide effector *NLP4* which is involved in programmed cell death in plant tissue (36) (Fig. 4*D*). To test whether the identified effectors are also derepressed in a *cer7* mutant, we generated *BAS113-RFP* and *BAS3-RFP* gene fusions and transformed them into the *cer7* mutant strain and Guy11, respectively. We observed high levels of Bas3-RFP and Bas113-RFP fluorescence in conidia of the *cer7* mutant transformants, compared to Guy11 (Fig. 4*E*), which provides further evidence that Rgs1 regulates additional effectors to Mep2. Transcriptional profile analysis of the 60 putatively Rgs1-repressed effectors during infection demonstrated that they peak in expression at either 48 or 72 hpi (*SI Appendix*, Fig. S7*B*).

### Rgs1 Contributes to Pathogen Fitness during Rice Blast Disease.

**Fig. 5.**
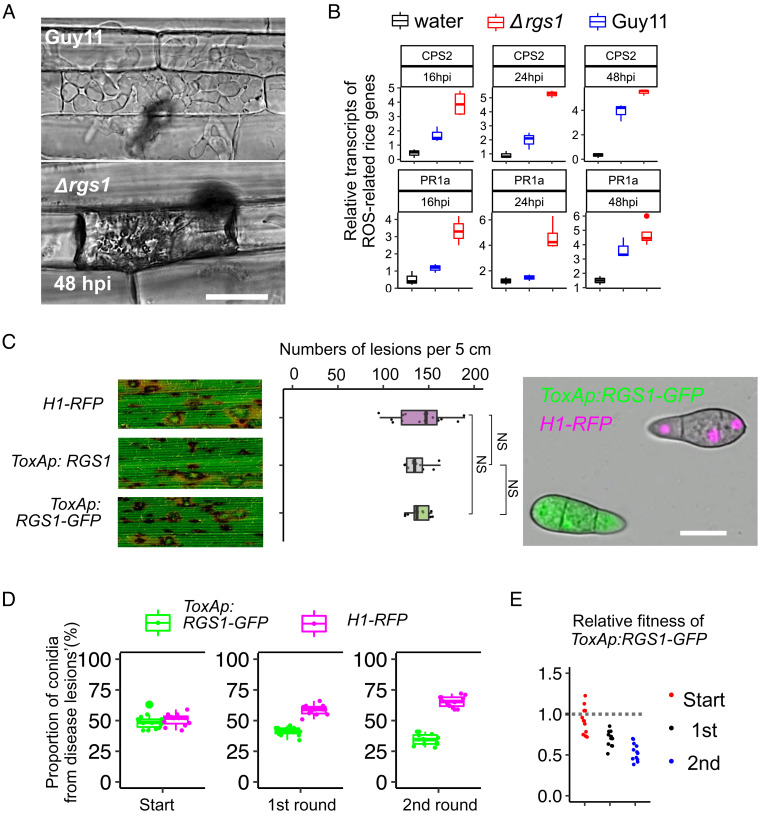
Rgs1 prevents rice defence gene expression and is a fitness determinant for rice blast disease. (*A*) Micrographs showing growth of invasive hyphae of Guy11 and Δ*rgs1* 48 hpi. (Scale bar, 10 µm.) Rice leaf sheaths of rice cultivar CO39 were inoculated with conidial suspensions of Guy11 and Δ*rgs1*. (Scale bars, 12 μm.) (*B*) Boxplots to show fold change values as relative transcripts of defence-related genes *CPS2* and *PR1a* in rice. Rice leaf sheath samples were inoculated with 0.02% gelatin (black), conidial suspension of Guy11 (blue) and Δ*rgs1* (red). The qRT-PCR was performed using rice housekeeping genes eEF1A and UBQ5 as standards. (*C*) Seedlings of rice cultivar CO-39 were inoculated with conidial suspensions of equal concentration (1 × 10^5^ conidia/mL) of *ToxAp:RGS1*, *ToxAp:RGS1-GFP,* and Guy11:H1-RFP. The boxplot represents the number of rice blast disease lesions per 5 cm in two independent repetitions of the experiment. Unpaired Student’s *t* tests were performed to determine significant differences. NS = no significant difference. Micrographs show the fluorescence signals of ToxAp:Rgs1-GFP and H1-RFP strains used in the relative fitness assay (Scale bar, 10 µm.) (*D*). Boxplots showing the number of spores recovered from disease lesions following mixed infections with conidial suspensions of *M. oryzae* strains expressing *H1-RFP* and *ToxAp:RGS1-GFP* respectively. After 7 d, spores were collected from disease lesions, the ratio of each genotype determined and then used for subsequent inoculation of CO-39 seedlings. (*E*) The relative fitness of the *ToxAp:Rgs1-GFP M. oryzae* strain was carried out using the formula: Relative fitness = x_2_(1 − x_1_)/x_1_(1 − x_2_), where x_1_ is the initial frequency and x_2_ the final frequency.

Given that Rgs1 potentially regulates the expression of a large group of effectors, we decided to investigate the biological significance of the effector regulation mediated by Rgs1. When we inoculated rice leaf sheath with conidia of Δ*rgs1*, we found that invasive hyphae grew more slowly than those of Guy11, being largely restricted to the initial epidermal cell colonized at 48 hpi (Fig. 5*A*). We therefore tested whether reduced growth in plant tissue was a consequence of the misregulation of effector gene expression. As many effectors act to suppress plant defence, we performed a qPCR experiment to investigate expression of a subset of defence-related rice genes from leaf sheath samples following infection by Guy11 and Δ*rgs1*, respectively. Transcripts of rice *PR1a* (37) and *CPS2* (38), are induced significantly at 16 hpi and at 24 hpi in rice tissue inoculated by strain Δ*rgs1*, compared to infection with Guy11 or noninfected tissue (Fig. 5*B*). These results are consistent with Rgs1 being required for the correct temporal dynamics of effector gene expression. However, we recognized that the role of Rgs1 was most likely to be involved in repressing effector gene expression prior to plant infection and therefore decided to investigate the consequence of overexpressing *RGS1* throughout plant infection. For this, we generated *M. oryzae* strains carrying *ToxA:RGS1-GFP* that constitutively express *RGS1* approximately fivefold higher than when driven by its native promoter (*SI Appendix*, Fig. S8*A*). Overexpression of *RGS1* did not lead to changes in phenotypes associated with the G-protein regulation function of the protein, such as aerial hyphal growth, or appressorium development on hydrophobic or hydrophilic surfaces when compared to Guy11 (*SI Appendix*, Fig. S8*B*). However, overexpression of *RGS1* did lead to complete repression of Mep2-GFP fluorescence during invasive growth with no visible BIC localization observed (*SI Appendix*, Fig. S8*C*). To test the role of the N-Rgs1 domain specifically, we independently expressed *ToxAp:N-RGS1* and found that overexpression of the N-terminal domain also did not affect G-protein–related functions of Rgs1, but did prevent Mep2 expression and BIC localization during plant infection (*SI Appendix*, Fig. S8*C*). Furthermore, qRT-PCR analysis revealed that transcripts of *MEP2, BAS3, BAS113*, and *MEP19* did not accumulate during *in planta* growth by the Rgs1 or N-Rgs1 overexpression lines of *M. oryzae* (*SI Appendix*, Fig. S8*D*). Standard spray infections did not reveal any difference in the ability Rgs1 or N-Rgs1 overexpression strains of the fungus (Fig. 5*C*) (*SI Appendix*, Fig. S8*C*). We reasoned that this assay might not be sufficiently sensitive to detect small differences in the ability of these strains to cause blast disease. We therefore used a recently described relative fitness assay to evaluate the effect of overexpressing Rgs1 (25). For this, we used a mixed spore inoculum of the *ToxAp:Rgs1-GFP* strain and a wild-type Guy11 strain expressing H1-RFP, which can be distinguished because it has red nuclei. The fluorescent markers are simply used as a means of visually distinguishing conidia of each strain. Before conducting the assay, we confirmed the two strains did not any differences in conidiogenesis (*SI Appendix*, Fig. S9*A*), as the assay relies on this trait to measure the ability to complete the pathogenic life cycle (25). Conidia were mixed in a 1:1 ratio and used to inoculate CO-39 seedlings (Fig. 5*D*). We allowed disease symptoms to develop and then recovered conidia from lesions at 7 dpi. We recorded the ratio of each spore type (green or with red nuclei, respectively) recovered from disease lesions and then carried out a second round of infection using the same ratio. After checking, there was no impaired appressorium formation for the spores (*SI Appendix*, Fig. S9*C*). We observed that the proportion of *ToxAp:RGS1* conidia reduced after two generations of infection to 30.83% and showed a fitness coefficient of 0.45 after two generations (Fig. 5*E*). We observed that the Rgs1 overexpression strain was able to colonize rice tissue and that conidia from each generation could form appressoria normally (*SI Appendix*, Fig. S9 *B* and *C*). Rgs1 overexpression strains are however significantly under-represented in the pathogen population after two generation of mixed infections. We conclude that overexpression of *RGS1* has an important fitness consequence at a population level, consistent with its action as a transcriptional regulator of genes associated with biotrophic fungal growth.

## Discussion

One of the hallmarks of fungal effector proteins is that they are expressed specifically during pathogenesis. Indeed, one of the main strategies for identifying putative effector candidates has been to use transcriptional profiling to identify differentially expressed pathogen genes. This has revealed the presence of temporally coexpressed families of effector genes in many fungal pathogens including *Ustilago maydis* (39) and *M. oryzae* (25). In rice blast, a very large repertoire of 546 effector genes has been reported, which are temporally coexpressed throughout invasive growth between 8 h and 144 h after infection, with large families of structurally related effectors coexpressed between 24 h and 48 h after infection during biotrophic proliferation of the fungus in rice tissue (25). The complexity of the spatiotemporal regulation of effector gene expression—in which cytoplasmic effectors localize specifically at the BIC during invasive hyphal growth—suggests that sophisticated transcriptional regulation must be present to ensure that effectors are produced and deployed at the correct time and in the correct cells. But even though effectors have been studied intensively in *M. oryzae*, leading to insight into their host targets and structure–function relationships (40, 41), very little is known about effector gene regulation. In contrast to bacterial pathogens, where effector gene regulation is well understood and associated with coordinate operon-based gene control (42), there have been only a limited number of regulators of fungal effector expression identified to date (27, 30).

In this study, we developed a simple forward-genetic screen to identify potential regulators of effector gene expression in *M. oryzae*. Using a reporter line of *M. oryzae*, we selected mutants showing constitutive expression of an effector-encoding gene, by simply picking mutants with green fluorescent spores—a stage of development when effectors are not normally expressed. Using whole-genome sequencing and bulked segregant analysis, we identified Rgs1, a well-known regulator of G-protein signaling during appressorium development, as being necessary for expression of the effector-encoding gene *MEP2*. Rgs1 is necessary for the repression of transcription of a group of effector-encoding genes in conidia, that are among a group of 697 genes which are Rgs1-dependent. This repression is clearly important biologically, as overexpressing Rgs1 during plant infection leads to a reduction in pathogen fitness.

Rgs1 is one of a group of 8 RGS proteins in *M. oryzae* that play distinct and overlapping regulatory functions (17). Rgs1 is involved in mediating perception of a hard, hydrophobic surface to induce appressorium morphogenesis. The noncanonical G-protein coupled receptor (GPCR), Pth11, responds to this inductive cue, leading to dissociation of the Ga subunit, MagA, from the heterotrimeric Gabg complex (17). MagA activity is regulated by Rgs1, to control the levels of cAMP, leading to activation of the cAMP/protein kinase A pathway (17, 33). In addition, Rgs1 negatively regulates the Ga subunit protein, MagB, during asexual conidiation (15) and mating (16). Rgs1 is phosphorylated by casein kinase 2 at the plasma membrane and late endosome, which is essential for its GTPase-activating protein (GAP) activity (17). RGS proteins are well known to mediate GPCR signaling functions due to DEP-domain–mediated tethering (43), which is consistent with the tandem DEP domains found in Rgs1 (33). An important question arising from this work is how an RGS protein, such as Rgs1, could also exert a transcriptional regulator function. It is not clear, for instance, whether this is a direct function of Rgs1 or whether the RGS protein regulates expression of additional transcription regulators via a cell signaling function. Previously, it was reported that Rgs1 undergoes endoproteolytic cleavage to produce the DEP–DEP protein N-Rgs1, and the RGS catalytic protein C-Rgs1 (15). This mirrors the endoproteolytic cleavage of the Sst2 RGS protein in *Saccharomyces cerevisiae*, which has been proposed to serve a regulatory function of its RGS catalytic activity (43, 44). Interestingly, we found evidence in RNA-seq data from a time course of rice infection of a potential exon skipping event that may occur in *M. oryzae*, suggesting that N-Rgs1 is preferentially generated during the prepenetration stage of development. Furthermore, the DEP–DEP N-Rgs1 protein is able to complement the Δ*rgs1* mutant phenotype with respect to Mep1 regulation, while the RGS C-Rgs1 cannot. Additionally, Rgs1-insensitive alleles of MagA, MagB, and MagC, GTPase-inactive or constitutive alleles of MagA and MagB, and even Δ*magA, ΔmagB,* or Δ*magC* null mutants showed no effect on the effector regulatory ability of Rgs1. When considered together, this is consistent with a model whereby the effector regulatory function of Rgs1 resides independently within N-Rgs1 and is associated with the prepenetration stage, when Rgs1 regulates MagA and cAMP levels during appressorium morphogenesis (15, 17, 33). Interestingly, N-Rgs1 shows activity in a yeast transactivation assay, but the absence of DNA-binding activity in N-Rgs1, suggests that if the protein does have a transcriptional regulatory function, then it must act in association with other proteins. There are examples of RGS proteins exerting transcriptional functions (45). In humans, for example, RGS2, RGS10, and RGS12, are nuclear proteins, while RGS4, RGS14, and RGS16 are nucleocytoplasmic shuttle proteins (46, 47). Human RGS6 is, furthermore, subject to complex alternative splicing with 36 distinct splice variants present that either localize to the cytoplasm, nucleus or nucleolus of neurons in the brain (48). RGS12, meanwhile, has been shown to have transcriptional activity, which resides solely in an N-terminal domain which can act as a transcriptional repressor and also has cell cycle-regulating activities, independent of its RGS domain (45). In our experiments, Rgs1 appears to localize to the cytoplasm predominantly with some punctate distribution also observed, although N-Rgs1 has also been reported to show endomembrane/vesicular localization (33). It is therefore possible that Rgs1 interacts with the plasma membrane or endomembrane compartments via its DEP domains and exerts a signaling function that ultimately activates a downstream transcription factor (49). The RGS protein FlbA in *Aspergillus niger*, for example, involved in regulation of sporulation regulates expression of many downstream functions via a set of transcription factors, including rpnR which regulates protein secretion and stress responses (50), and Fum21 which regulates production of the mycotoxin fumonisin (51). We are currently screening putative interactors of N-Rgs1 to identify its potential mode of action in the regulation of effector function and investigating whether a subpopulation of N-Rgs1 might enter the nucleus. Rgs1 is important for correct temporal expression of a diverse group of effectors, because Δ*rgs1* mutants show a reduction in virulence, suggesting that in addition to the well-known G-protein–associated functions of the protein, its mistiming of effector expression may contribute to reduced virulence. Overexpression of Rgs1 or the N-Rgs1 domain alone—which repressed the expression of a larger group of effectors but did not affect G-protein associated Rgs1 functions—also reduced the relative fitness of *M. oryzae* at a population level, consistent with a requirement for the concerted action of Rgs1-dependent effectors to fungal virulence.

In summary, we have demonstrated a successful way to identify a novel regulator of effector gene expression. By using a simple forward genetic screen, it has proven possible to identify a regulator that ensures the correct temporal expression profile of a large group of at least 60 effector genes, preventing their premature expression prior to plant infection. This genetic approach has therefore revealed an unexpected link between the developmental biology of the prepenetration stage of *M. oryzae*, regulated by the RGS protein Rgs1, and events that occur after host cell invasion, including the expression of fungal effectors that suppress plant immunity and contribute to rice blast disease.

## Materials and Methods

### Fungal and Plant Growth Conditions.

Growth and maintenance of *M. oryzae* isolates, media composition, nucleic acid extraction, and fungal transformation were all performed as previously described (52). Strains were collected from stocks and inoculated to solid complete medium (CM) and incubated at 24 °C with a 12-h light/dark cycle. For plant infection, conidial suspensions of *M. oryzae* at 5 × 10^4^ conidia mL^−1^ in 0.2% gelatin were spray inoculated onto 21-d-old rice seedlings of CO39 using an artist’s airbrush. Rice blast symptoms were scored using disease lesion density 5 d postinoculation, as described previously (52). DNA amplification, cDNA synthesis, and qRT-PCR were carried out using standard procedures with specific primers (*SI Appendix*, Table S1). Further details regarding *M. oryzae* mutant generation, UV mutagenesis, and bulked segregant analysis are provided in *SI Appendix*, *SI Materials and Methods*.

### Appressorium Development Assays and Leaf Sheath Infection.

Appressorium assay of rice blast fungus was performed as previously described (35). Briefly, conidial suspensions were prepared at 5 × 10^4^ conidia mL^−1^ in water, and 20 µL of the suspension placed onto the surface of a hydrophobic coverslip (Corning) and incubated in a controlled environment chamber at 24 °C. Live cell imaging of plant penetration, effector localization in invasive hyphae and growth of the fungus in planta used a leaf sheath assay, described previously. Briefly, a suspension of 5 × 10^4^ conidia mL^−1^ was prepared in 0.2% gelatin and inoculated into the hollow space of the leaf sheath tissue that was dissected from the flag leaf of 21-d-old rice seedlings of cultivar CO-39. A single epidermal layer of the leaf sheath was trimmed and mounted for microscopy (24).

### Live Cell Imaging and Quantitative Analysis.

To screen the mutant, Sterile water was used to harvest spores from a 7-d-old *M. oryzae* CM plate culture, before filtering through sterile Miracloth (Calbiochem). A 20 µL aliquot of suspension was transferred to a microscope slide and covered with a clean cover slip before imaging with epifluorescence and differential interference contrast (DIC) microscopy. For epifluorescence and differential interference contrast (DIC) microscopy, samples were observed using an IX-81 inverted microscope (Olympus) and a UPlanSApox100/1.40 oil objective. Images were captured using a photometrics CoolSNAP HQ2 camera system incorporated with MetaMorph software packages (MDS Analytical Technologies, Winersh, UK). Epifluorescence parameters for GFP were excitation 488 at nm, and RFP excitation at 561 nm. Quantification of fluorescence intensity was performed using Fuji-ImageJ (53). To measure fluorescence intensity, conidia of interest were selected using drawing tools. “Measure” was used to obtain the “Integrated Density” (ID) for the conidial fluorescence signal. The background region next to the conidium of interest was selected and measured to obtain the pixel value for “mean fluorescence of background readings” (MFBR). The values of conidia and background were extracted and saved to calculate the corrected cell fluorescence (CCF) using the formula: CCF = Integrated Density (ID) - mean fluorescence of background readings (MFBR). Details of RNA extraction, RNA-seq analysis, quantitative real-time PCR, yeast transactivation assay, and one-yeast hybrid system are given in *SI Appendix*, *SI Materials and Methods*.

## Supplementary Material

Appendix 01 (PDF)

## Data Availability

RNA-seq data of *M. oryzae* and the genome sequence of *cer7* mutant strain reported in this paper were deposited in the European Nucleotide Archive - EMBL-EBI database under accession number: PRJEB45710 (54).
