# Supplementary material for: Rgs1 is a regulator of effector gene expression during plant infection by the rice blast fungus Magnaporthe oryzae
Source: Proc Natl Acad Sci U S A. 2023 Mar 13;120(12):e2301358120. doi: 10.1073/pnas.2301358120 (PMC10041150; doi:10.1073/pnas.2301358120)
Supplement: Supplementary file 1 — Appendix 01 (PDF) [file pnas.2301358120.sapp.pdf]

PNAS

Supplementary Information for

Rgs1 is a regulator of effector gene expression during plant infection by the rice blast fungus *Magnaporthe oryzae*

Bozeng Tang, Xia Yan, Lauren S. Ryder, Mark Jave A. Bautista[a], Neftaly Cruz-Mireles, Darren M. Soanes, Camilla Molinari, Andrew J. Foster, and Nicholas J. Talbot

Nicholas J. Talbot
Email: nick.talbot@tsl.ac.uk

This PDF file includes:

SI Materials and Methods
Figures S1 to S9
Table S1
Table S2
Table S3
SI References

SI Materials and Methods

Generation of *M. oryzae* mutants and strains expressing GFP or RFP fusions

To generate *M. oryzae* transformants expressing Mep2-GFP, the full-length coding sequence of *MEP2* (1) was cloned with 1.5 kb of upstream promoter sequence and fused at its C-terminus to eGFP. The Mep2-GFP plasmid was constructed using the linearized pCB1532 carrying the acetolactate synthase gene which confers resistance to sulfonylurea. Independent *M. oryzae* transformants were used for screening and selected on medium with sulfonylurea (400 µg mL^{-1}) for fluorescence localization and quantification. In-Fusion cloning (Clontech Laboratories) was used to generate *RGS1*^{WT}, *rgs1*^{cer7}, N-Rgs1, C-Rgs1, Bas113-RFP, Rgs1-GFP, Bas3-RFP, *MagA*^{G187S}, *MagB*^{G183S}, *MagA*^{Q208L}, *MagB*^{Q204L}, *MagC*^{G184S}, *MagB*^{G42R}. *MagA*^{G187S}, *MagB*^{G183S}, and *MagC*^{G184S}, ToxAp:Rgs1-GFP, ToxAp:Rgs1, and ToxAp:N-Rgs1 strains, using primers in Table S1. *RGS1*^{WT}, *rgs1*^{cer7}, N-Rgs1, C-Rgs1, were cloned under control of the *RGS1* native promoter (1 kb) and native terminator (600bp) sequence, except N-Rgs1 which was fused to the TrpC terminator, Bas113-RFP, Rgs1-GFP, Bas3-RFP, and ToxAp:Rgs1-GFP were cloned with their full protein coding sequences without stop codons driven by their native promoters, except *ToxAp:Rgs1-GFP* which was fused with ToxA promoter at its N-terminus. The fragments were then fused with eGFP in-frame at the C-terminus. *MagA*^{G187S}, *MagB*^{G183S}, *MagA*^{Q208L}, *MagB*^{Q204L}, *MagC*^{G184S}, *MagB*^{G42R}. *MagA*^{G187S}, *MagB*^{G183S}, and *MagC*^{G184S} were cloned with their native promoters and point mutations generated as described previously (2). Constructs were routinely cloned into a SpeI and NotI digested StrataClone vector, pSC-A-amp/kan, with the BAR gene conferring bialophos resistance (3, 4). Plasmids were then introduced into *M. oryzae* by fungal transformation (5) and transformants selected by growing on CM supplemented with glufosinate (30 µg mL^{-1}). All transformants were evaluated by PCR using primers flanking the predicted insertion to select those carrying single plasmid insertions. This was subsequently verified by whole genome sequencing.

To generate the strains *Δrgs1, ΔmagA/cer7, ΔmagB/cer7, ΔmagC/cer7*, and native allelic replacement of *RGS1* with the hygromycin phosphotransferase gene cassette (HYG), bestowing hygromycin B resistance, the split-marker strategy were performed as previously described with minor modifications (6-8). Briefly, we first amplified the 1 kb *HY* and 0.7 kb length YG fragments using the primers M13F and HY split for *HY*, M13R and YG split for *YG* (6). To create the fusion in the first round of PCR, a 3.2 kb length fragment of DNA sequence of the mutant allele of *RGS1*^{cer7} carrying the identified SNP, including 600 bp downstream sequence (3'-UTR), was amplified using allele-specific primers (P1 and P2). The 5' end of primer P1 is 97 bp upstream of the SNP. The 5' end of primer P2 included an extension overhanging sequence complementary to the M13F primer site. A 1.4 kb of DNA fragment was amplified from the right flank of the *RGS1* gene from *cer7* using allele-specific primers (P3 and P4). An overhanging sequence was added to the 5' end of primer P3, complementary to the M13R sequence. The 5' end of the right flank is 100 bp downstream of

the 3'-UTR. Isolated genomic DNA of the *cer7* strain was used as template for the PCR. After amplification the two initial PCR products were mixed with *HY* and *YG*, respectively, and used as templates for a second round of PCR. During the second round PCR, primers P1 and *HY* split were used to amplify a 4.2 kb length fragment, and primers P4 and *YG* split were used to amplify a 2.1 kb length fragment. These products were gel-purified, mixed in equimolar concentrations and transformed into the *Mep2-GFP* strain of *M. oryzae*. The fragments were used to replace the original wild type *RGS1* allele, based on three crossover events (7). The joined *HYG* allele confers hygromycin B resistance and allowed transformants to grow on CM plates supplemented with hygromycin B. We obtained 25 hygromycin B resistant transformants, isolated genomic DNA from these and performed PCR to amplify the fragment from 5' of left flank of *RGS1* gene locus to 3' of right flank, using primers P1 and P4. Targeted allelic replacement transformants had an additional 1.5 kb sequence, generating an 8.0kb amplicon, while unsuccessful transformants still contained the wild type *RGS1* allele on a 6.5 kb amplicon. Transformants were selected on complete medium (CM) (1) supplemented with hygromycin B (200 µg mL^{-1}) and assessed by PCR to amplify the specific allele which was then sequenced to ensure the *cer7* SNP was present. All mutants and transformants generated in this study are listed in Table S2.

UV mutagenesis of *M. oryzae*

Conidia of *M. oryzae* were harvested from a CM plate culture using a sterile plastic spreader with 2 mL sterile water. Conidial suspensions were generated in 200 µL sterile distilled water containing 0.1% Tween 20, at a concentration of 5 $x10^5$ conidia mL^{-1} before spreading onto the surface of modified CM agar containing 1% sorbose. The Petri dish was exposed without its lid under UV light at 254 nm using a UV crosslinker (Stratagene) in the dark. A calibration test was first performed to estimate the time necessary to result in either a 40% or 90% kill rate. After UV mutagenesis, plates were immediately wrapped in aluminium foil to avoid DNA photo-repair after ultraviolet light-induced DNA damage. Plates were then incubated at 26°. After 2-3 days, the foil was removed and the plates were grown for a further 4-5 days. Putative mutants were selected based on expression of Mep2-GFP using an epifluorescence stereo microscope Leica M205 FCA. The candidate mutants were then collected using a sterile needle onto fresh CM agar and incubated for 5 days. To perform single spore isolation for each potential candidate mutant, a conidial suspension from each plate was sprayed onto 4% water agar and four single conidia were individually removed using a sterile mounted needle. The resulting single conidia were transferred onto 48 well plates containing CM. The purified mutant strains were then screened for expression of Mep2-GFP as described above and stored as filter paper stocks at -20°C.

Bulked segregant analysis, SNP calling and alternative splicing analysis

Bulked segregant analysis (BSA) was performed, as previously described with minor modifications (9). Briefly, the *cer7* mutant strain (*Mat1-1*) and wild-type strain TH3 (*Mat1-2*) were grown at 24°C for 7 days on oatmeal agar medium, and then at 18°C until flask-shaped perithecia were visible. After 4-6 weeks growth, mature perithecia were removed and transferred onto 4% water agar medium. The collected perithecia were crushed with a sterile needle to release asci. Mature asci were removed and ascospores dissected under a Leica M205 FCA stereo microscope using a micro-manipulator (Singer Instruments). Collected ascospores were transferred individually to a 48-well plate containing CM and incubated for 5 days (10). Progeny were next screened by phenotypic assessment of fluorescence. Genomic DNA was then extracted from ascospore progeny, as described previously (5). Equal amounts of DNA from each progeny (100ng per progeny) were then bulked. DNA samples were bulked into two samples: wild-type progeny showing low expression of Mep2-GFP, and mutant progeny showing highly expressed Mep2-GFP. The two samples were sequenced using an Illumina HiSeq 2500 generating 125-bp paired-end sequences (University of Exeter Sequencing Service). Low-quality reads were filtered using Trimmomatic (11). SNP calling analysis was performed, as described previously with minor modifications (9). Filtered reads were mapped to the *M. oryzae* 70-15 reference genome (fungi.ensembl.org/Magnaporthe_oryzae/) using Bowtie2 with default parameters (12). Varscan (13) was then used to discover variants by inputting BAM files generated from alignment of reads of the genome sequence of the *cer7* mutant, pooled BSA samples, and the parental Mep2-GFP strain of Guy11 used for mutagenesis. To discover SNPs, filtering was applied based on a minimum read depth of 50 and minimum base identity of 95%. The Integrative Genome Viewer (IGV) (14) was used to manually inspect variants from read alignment for evidence of mutations by input of corresponding bam files.

For alternative splicing analysis, BAM files were generated using RSubread (v.2.10.5) with default settings (15). Putative alternative splicing events of *RGS1* were analyzed based on transcript read densities and visualized using IGV software (14). Peptide sequences of all coding genes in version 8.0 of *M. oryzae* genome sequence were used to predict effectors by EffectorP-2.0 as described (1). Verification of the insertion site of *MEP2-GFP* at a single site was verified by whole genome assembly. Raw reads were aligned to the full plasmid sequence of pCB1532 using Bowtie2 v2.2.9. The sorted BAM file was loaded into the IGV genome viewer to visualize the coverage of raw reads across the Mep2-GFP sequence. The resulting reads aligned to the plasmid and flanking region of the plasmid insertion site were extracted. The total raw reads were realigned against the extracted reads using Exonerate v2.2.0 (16), showing at least 50 bp alignment without any gap, representing reads located at the junction between the inserted plasmid and *M. oryzae* genome. The resulting reads were then aligned against *M. oryzae* genome using Exonerate v2.2.0 and the location of insertion of plasmid visualized via IGV to confirm that the reads aligned to a single locus in Chr1 as a single copy insertion.

RNA extraction, RNA-seq analysis and quantitative real-time PCR

Conidia from *cer7*, *Δrgs1*, and Guy11 were harvested from 7-day old CM agar plates. Total RNA was extracted from conidia using the Qiagen RNeasy Plant Mini kit according to the manufacturer's instructions. RNA-seq libraries were prepared using 10 µg of total RNA for Illumina HiSeq 2500 generating 150-bp paired-end sequences (Novogene, Beijing). Low quality reads were filtered by Trimmomatic v0.32 prior to the alignment against version 8.0 of *M. oryzae* genome sequence using bowtie2 (version 2.3.4.3). Quality of alignment was assessed by qualimap2 (version 2.2.1). Pearson correlation and principal component analysis was performed to determine correlation between the samples. The sva package was used to remove batch effects (17). Differentially expressed genes were determined by DESeq2 through a threshold of log2FC and adjusted p-values ($p<0.05$). for enrichment analysis, Clusterprofiler was used to performed KEGG metabolic pathway enrichment analysis (18). For qRT-PCR, an Affinity Script QPCR cDNA Synthesis Kit was used to generate cDNA, according to manufacturer's instructions. Briefly, a reaction premixed with 10 µL First-Strand Master Mix, 3µL of oligo(dT) primer, 1 µL of AffinityScript RNase Block enzyme mixture, and 3 µg total RNA, was prepared and incubated at 25 °C for 5 min. The sample was incubated at 42 °C for 15 min to allow cDNA synthesis, before further incubation at 95 °C for 5 min to terminate the cDNA synthesis reaction. Quantitative real-time PCR (qPCR) analysis was conducted using the Stratagene Mx3000TM Real-Time PCR. Each reaction contained 1.25 µL of 606 a 1:5 (v/v) dilution of cDNA, 0.2 µM of each primer and 1X SYBR® Premix Ex Taq™ (Tli RNase H plus, RR420A, Takara) in a total reaction volume of 12.5 µL. PCR conditions were: 1 cycle of 1 min at 95°C; 40 cycles of 5 s at 95°C and 20 s at 60°C; and a final cycle of 1 min at 95°C, 30 s at 58°C and 30 s at 95°C for the dissociation curve. For measurement of the fold change, an efficiency corrected calculation model was made using the formula ((target) ΔCt target (control – sample)) / (housekeeping) ΔCt housekeeping (control – sample) (19).

Western blot analysis

To test *RGS1* levels in vegetative and invasive growth, total protein was extracted from mycelium and infected rice leaf sheath using *M. oryzae* strains *ToxAp:GFP* (Control) and *RGS1p:GFP-RGS1*. For vegetative growth, mycelium was prepared from CM shake cultures (125 rpm) at 24°C for 48 h. Mycelium was then filtered, washed in distilled water and frozen in liquid nitrogen. For invasive growth, leaf sheath inoculation assays were performed, as previously reported (20) and frozen in liquid nitrogen after 32 hpi. Frozen samples were ground to fine powder in liquid nitrogen using a pestle and mortar. The powder was mixed with 2 times weight/volume ice-cold extraction buffer (10% glycerol, 25 mM Tris pH 7.5, 1 mM EDTA, 150 mM NaCl, 2% w/v PVPP, 10 mM DTT, 1× protease inhibitor cocktail (Sigma), 0.1% Tween 20 (Sigma)), centrifuged at 10,000g at 4°C for 10–20 min, and the supernatant passed through a 0.45 µm Minisart® syringe filter. Proteins were separated by SDS-PAGE and transferred onto a polyvinylidene difluoride (PVDF) membrane using

a Trans-Blot turbo transfer system (Bio-Rad). PVDF membrane was blocked with 2% bovine serum albumin (BSA) in Tris-buffered saline and 1% Tween 20 (Sigma). GFP detection was carried using a GFP (B2):sc-9996 horseradish peroxidase (HRP)-conjugated antibody (Santa Cruz Biotechnology, Santa Cruz, CA). An anti-actin antibody was used as the loading control for fungal and plant protein. Pierce ECL Western Blotting Substrate (Thermo Fisher Scientific) was used for detection. Membranes were imaged using ImageQuant LAS 4000 luminescent imager (GE Life Sciences).

Yeast Transactivation Assay and Yeast One-Hybrid Analysis

The transcriptional activity and DNA-binding ability of N-Rgs1 and Rgs1 were tested in *Saccharomyces cerevisiae* strain Y2HGold. Sequences encoding N-Rgs1 and C-Rgs1 were amplified from cDNA derived from total RNA of Guy11 conidia. Fragments were cloned into pGBKT7 (BD) and pGADT7 (AD) vectors, respectively, by in-fusion cloning (In-Fusion Cloning kit; Clontech Laboratories). pGBKT7(BD)-N-Rgs1, pGBKT7(BD)-Rgs1 were used to test transactivation activity, and pGADT7(AD)- N-Rgs1, pGADT7(AD)-Rgs1 were used to test DNA-binding ability. The empty vectors were co-transformed and used to test the transcriptional activation as a negative control. The vectors pGBKT7-53 and pGADT7-T were used as the positive control. Empty un-linearized bait vector pGBKT7 and prey vector pGADT7 were used as negative controls. BKT variants and GAD variants were co-transformed into chemically competent Y2HGold yeast cell following the manufacturer's manual. Successfully transformed yeast colonies were inoculated in 2mL of liquid SD/-Leu/-Trp selection media for overnight growth at 30°C. The yeast samples were then used to produce dilutions of OD_{600} 1, 1^{-1}, 1^{-2}, 1^{-3}, and 1^{-4}, respectively. Cell suspensions in 10µL droplets of each dilution were spotted on a SD/-Leu/-Trp/-His to determine the transactivation activity and DNA-binding ability, after 72 h growth at 30°C.

For yeast one-hybrid experiments, the Matchmaker Gold Yeast One-hybrid System kit (Takara Bio, USA) was used according to manufacturer's instructions. Briefly, a 1 kb fragment upstream of the *MEP2* gene was prepared as the bait sequence and ligated into digested vector pAbAi, which carries the bAr gene (AUR-1C) that confers resistance to AbA (Aureobasidin A) (Takara Bio, USA). The p-AbAi-*MEP2* vector, and positive control vector p53-AbAi were digested with BbsI, and then transformed into yeast strain Y1HGold (21), respectively. Y1HGold [*MEP2p* / AbAi] and Y1HGold [p53 / AbAi] was screened by growing on SD/-Ura. The minimum inhibitory concentration of AbA (200 ng/mL) for the bait strain was determined by growing on SD/-Ura/AbA medium. The cDNA sequence encoding N-Rgs1 was amplified from total RNA by RT-PCR using primers with overhanging sequences the prey vector pGADT7. The plasmid pGADT7- was then transformed into bait yeast strain Y1HGold [*MEP2p*/AbAi], and Y1HGold[p53/AbAi], respectively. The yeast samples were used to produce dilutions of OD600 1, 1^{-1}, 1^{-2}, 1^{-3}, and 1^{-4}, respectively. SD/-Leu/AbA^{200} medium was used to test the *MEP2p*/N-Rgs1 interaction.

Pathogenicity *in planta* for Fitness assay of *M.oryzae*

A relative fitness assay was performed according to previous studies with minimum modification (1, 3). Conidial suspensions of ToxAp:Rgs1-GFP and H1-RFP *M. oryzae* strains were harvested from 5 day old CM plate cultures. The concentration of conidia for each strain was determined using a heamocytometer and conidia were then mixed in a ratio of 1:1 to a final concernation of $5x10^4$ conidia per mL in 0.2% (w/v) gelatin. An aliquot of 20 µL of the conidial suspension was then placed onto the surface of 4 week old rice seedlings of cultivar CO-39 in a leaf drop inoculation. At least 20 leaf drops were used for each round of the experiment. Rice blast infection proceeded for 6 days until the appearance of disease lesions. Leaves were then transferred under high humidity to induce sporulation from disease lesions for 48h. Recovered spores were imaged by epifluorescence microscopy to detect ToxAp:Rgs1-GFP and H1-RFP fluorescence and the ration of red:green fluorescence was calculates. The equivalent conidial ratio was then used to prepare fresh inoculum from plate cultures of each *M. oryzae* strain and used to infect another batch of CO-39 seedlings seedling. We repeated the procedure for the second generation and calculated the ratio of red:green conidia from disease lesions. The fitness coefficient was calculated as described previously (1, 3).

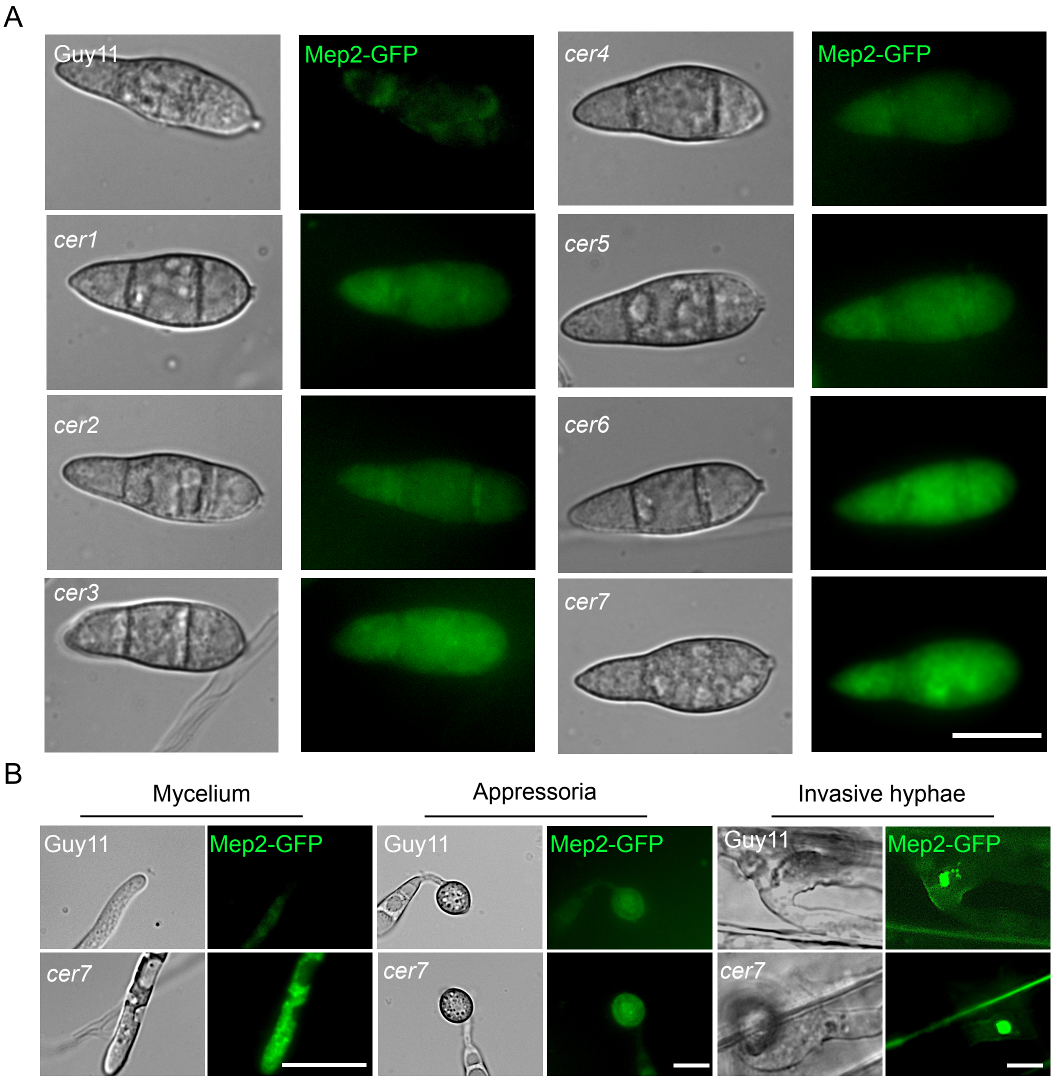

Fig. S1. Forward genetic screening identified mutants showing constitutive expression of Mep2-GFP. (***A***) Micrographs showing fluorescence of Mep2-GFP of conidia of 7 different mutants. Conidial suspensions from each strain were harvested from colonies after 5 days growth on CM plates and immediately visualized using epifluorescence microscopy. (Scale bar, 10 µm). (***B***) Expression of Mep2-GFP in the *cer7* mutant strain is constitutively activated during different development stages including mycelium, appressoria, and invasive hyphae. Micrographs showing fluorescence of Mep2-GFP in Guy11 and the *cer7* mutant, in mycelium, appressoria, and invasive hyphae. Mycelium was imaged after 24 hours growing in liquid CM culture. The image for appressorium development was captured 8hpi on a hydrophobic glass coverslip. Invasive hyphae were imaged 32hpi on CO39 rice leaf sheath inoculated with conidial suspensions at $1x10^4$/mL (Scale bar, 10 µm).

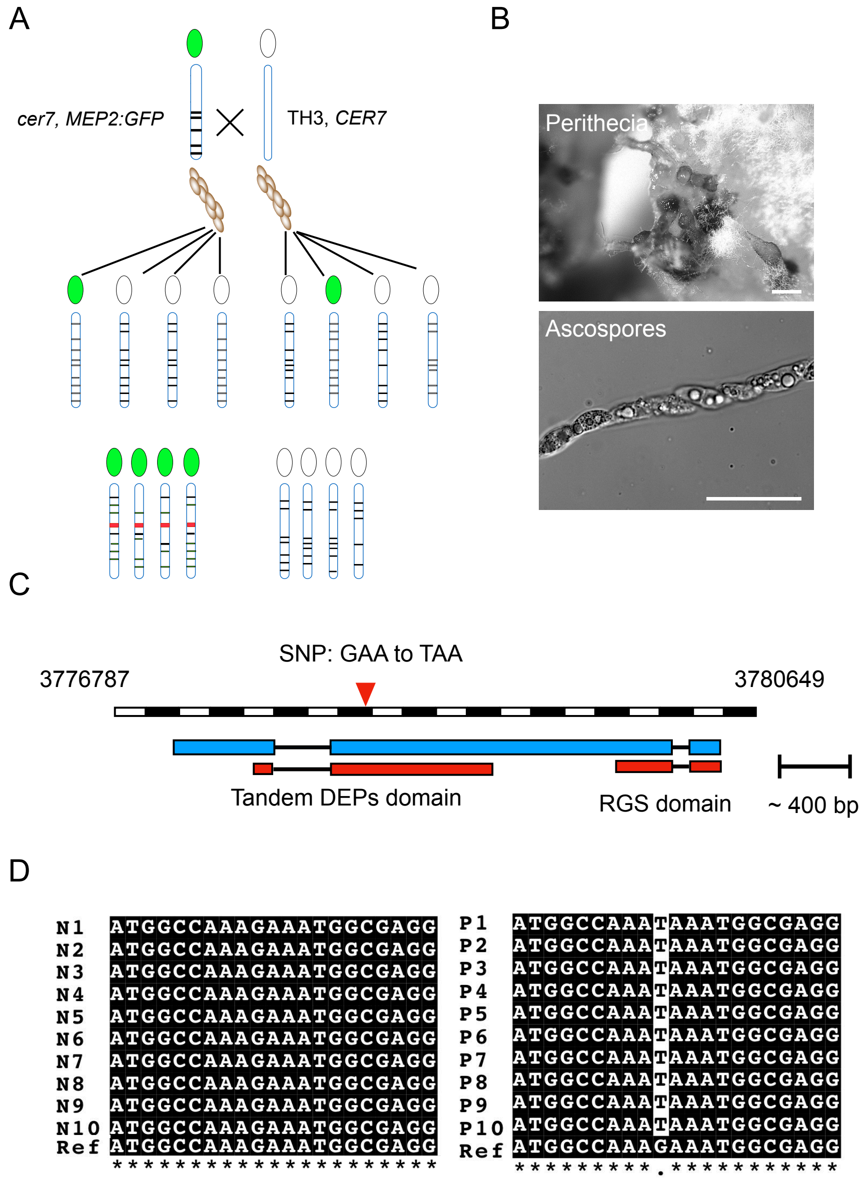

Fig. S2. Bulk segregant analysis defined a region of chromosome 2 showing very strong linkage to *cer7*. (***A***) Diagram to show bulked segregation analysis strategy to identify the mutant allele causing constitutive expression of *MEP2* in conidia. The *cer7* mutant (*Mat1-2*) and TH3 (*Mat1-1*) were used in sexual crosses by incubation together on oatmeal agar. After 4-6 weeks, perithecia developed and were collected and manipulated to release asci containing ascospore progeny. (***B***) Micrographs to show the perithecia and eight-spored ascus obtained from crossing *cer7* strain and TH3. (Scale bar, 10 μm). Images were captured after 8 weeks growth on oatmeal agar. (***C***) Overview of the genomic region of the *RGS1* locus to show transcript structures, and location of detected SNP in the highest linkage region identified by BSA. (***D***) Sequence analysis of amplicons of the *RGS1* coding sequences from 20 ascospore progeny. N1 to N10 are amplicons from progeny showing low Mep2-GFP fluorescence (wild-type), and P1 to P10 represent amplicons of progeny showing high expression of Mep2-GFP (*cer7*). "Ref" shows the DNA sequence of *RGS1* in the *M. oryzae* reference genome.

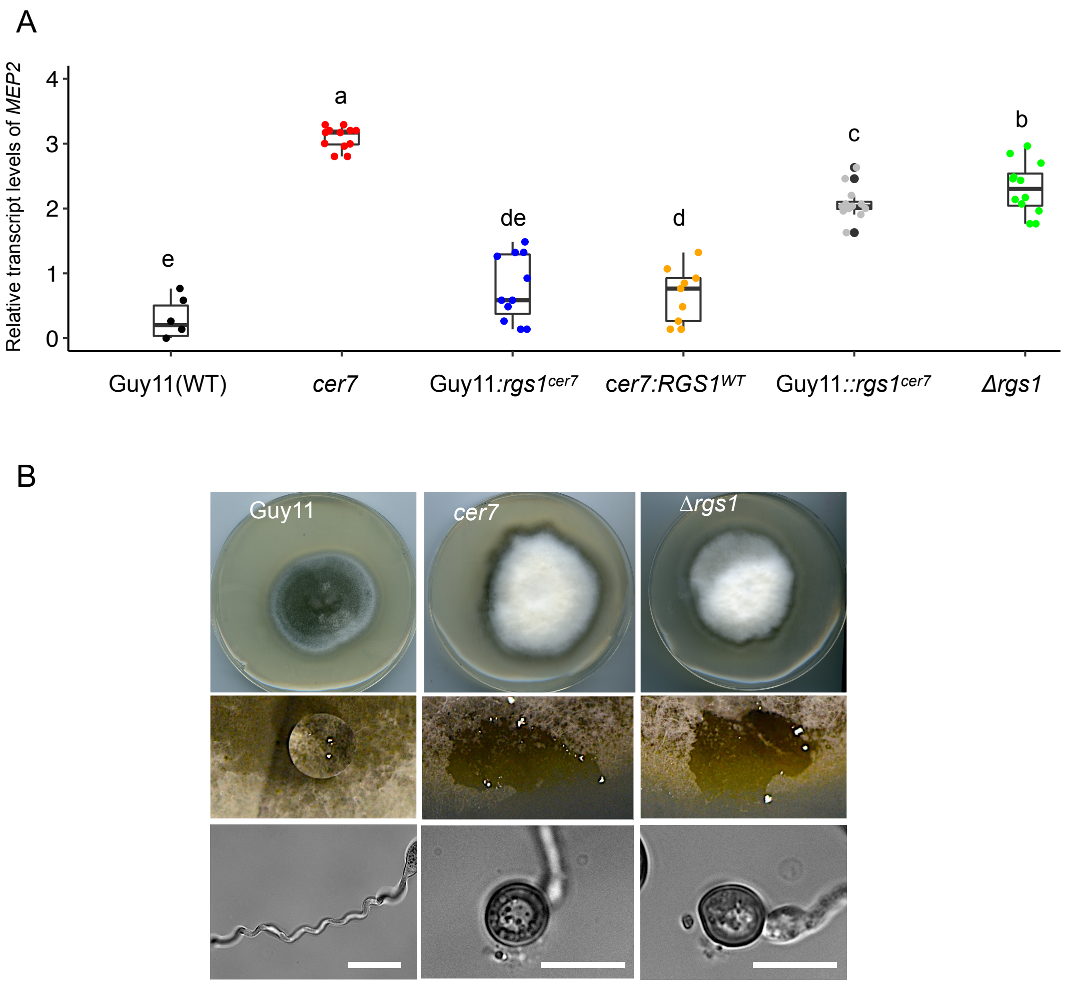

Fig. S3. Complementation analysis of the *cer7* mutant with *RGS1*. (***A***) Box plot to show fold change in relative transcripts of *MEP2* from mycelium of the wild type Guy11 (Black), *cer7* mutant (red), Guy11:$rgs1^{cer7}$ ectopic transformant (blue), *cer7:*$RGS1^{WT}$ complemented transformant (orange), Guy11::$rgs1^{cer7}$ allelic replacement mutant (grey) *Δrgs1* mutant (green). Expression was determined relative to the fungal actin gene. Letters refer to significant differences determined by One-way ANOVA tests ($p<0.05$, Duncan test). (***B***) Upper panel Images to show the colony morphology of wild type Guy11, compared to *cer7* and Δ*rgs1* mutants. Both *cer7* and Δ*rgs1* form white fluffy colonies when grown on complete medium. Mid panel images show droplets of water placed on the surface of plate cultures of Guy11, *cer7* and Δ*rgs1* mutant strains. Mutants show an easily-wettable phenotype. Photographs were taken 24hpi. Lower panel micrographs show appressorium development by *cer7* and Δ*rgs1* mutants on a non-inductive hydrophilic surface. (Scale bar, 10 μm).

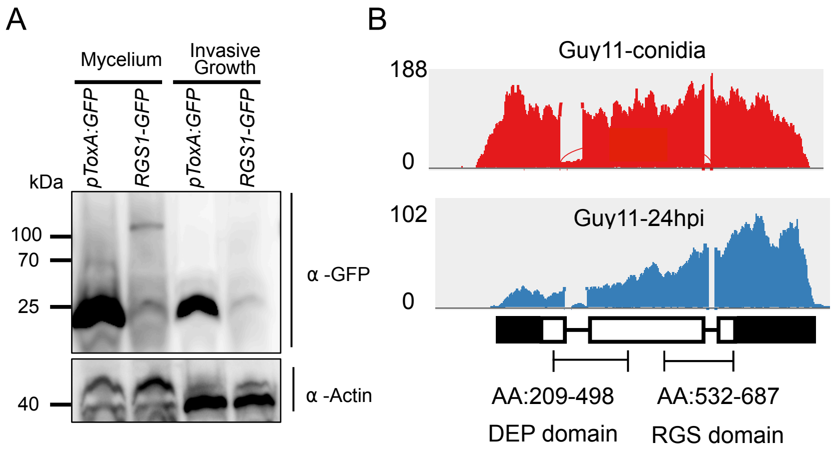

Figure S4. Expression analysis of *RGS1*. (***A***) Western blot showing abundance of Rgs1-GFP detected with anti-GFP antibody in mycelium and invasive hyphae (IH). Total protein extracted from mycelium of Rgs1-GFP after growing in liquid CM culture for 48 hours. Lysates were resolved by SDS-PAGE and analysed by western blot using antibodies against GFP. Detection using anti-actin was used as the loading control for fungal protein. Molecular mass standards in kDa are indicated on the left. (***B***) Sashimi plots showing mRNA transcript reads of *RGS1* in Guy11, as conidia at 0hpi (red), and during infection at 24hpi (blue). The bar plot demonstrated the depth of reads aligned to corresponding regions of the gene. RNA-seq from libraries of total RNA isolated from leaf-drop infection inoculated by wild-type Guy11 were used. The numbers on the left panel indicate the coverage of reads detected after alignment and visualized in IGV.

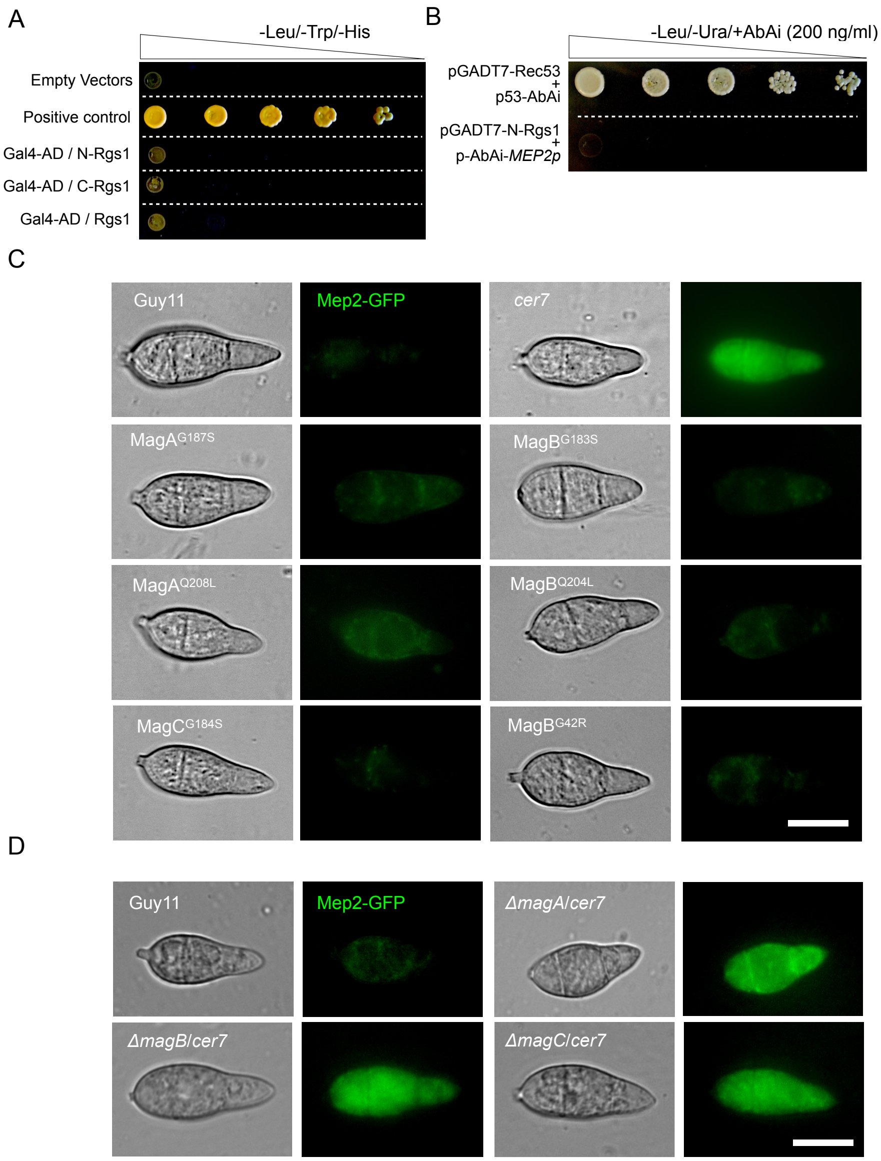

Fig. S5. (***A***) N-Rgs1 does not possess DNA-binding ability or bind to the *MEP2* promoter. DNA-binding assay of N-Rgs1, C-Rgs1, and full length Rgs1 in yeast cells. Co-transformation of Y2HGold strains was carried out with bait (BD) and prey (AD) vectors in the following combinations; pGBKT7 / pGADT7-N-Rgs1, pGBKT7 / pGADT7-C-Rgs1, pGBKT7 / pGADT7-Rgs1, and positive control (pGBKT7-53 and pGADT7-T) along with empty vectors. Cells were grown on double drop out or quadruple dropout medium. Images are representative of two biological replicates. (***B***) Yeast-one-hybrid assay to determine interaction between Rgs1 and upstream sequences of the *MEP2* gene. The Y1HGold strain containing p-AbAi-*MEP2p* was transformed by introducing plasmid pGADT7-Rgs1$^{1\text{-}498}$ and used to test growth on medium with -Leu/-Ura, supplemented with 200 ng/mL Aureobasidin A (AbA). (***C***) The repression of *MEP2* expression by Rgs1 occurs in a G-protein signalling-independent manner. Micrographs showing expression of Mep2-GFP in conidia of Guy11, *cer7*, MagA^{G187S}, MagB^{G183S}, MagA^{Q208L}, MagB^{Q204L}, MagC^{G184S}, and MagB^{G42R}. MagA^{G187S}, MagB^{G183S}, MagC^{G184S} (***D***) Conidia of *ΔmagA*/*cer7*, *ΔmagB*/*cer7,* and *ΔmagC*/*cer7* strains showing constitutive Mep2-GFP fluorescence. Images are representative at least 20 replicates examined for each experiment. Conidial suspensions from each strain were harvested from colonies after 5 days growth on CM plates and immediately visualized by epifluorescence microscopy. (Scale bar, 10 µm).

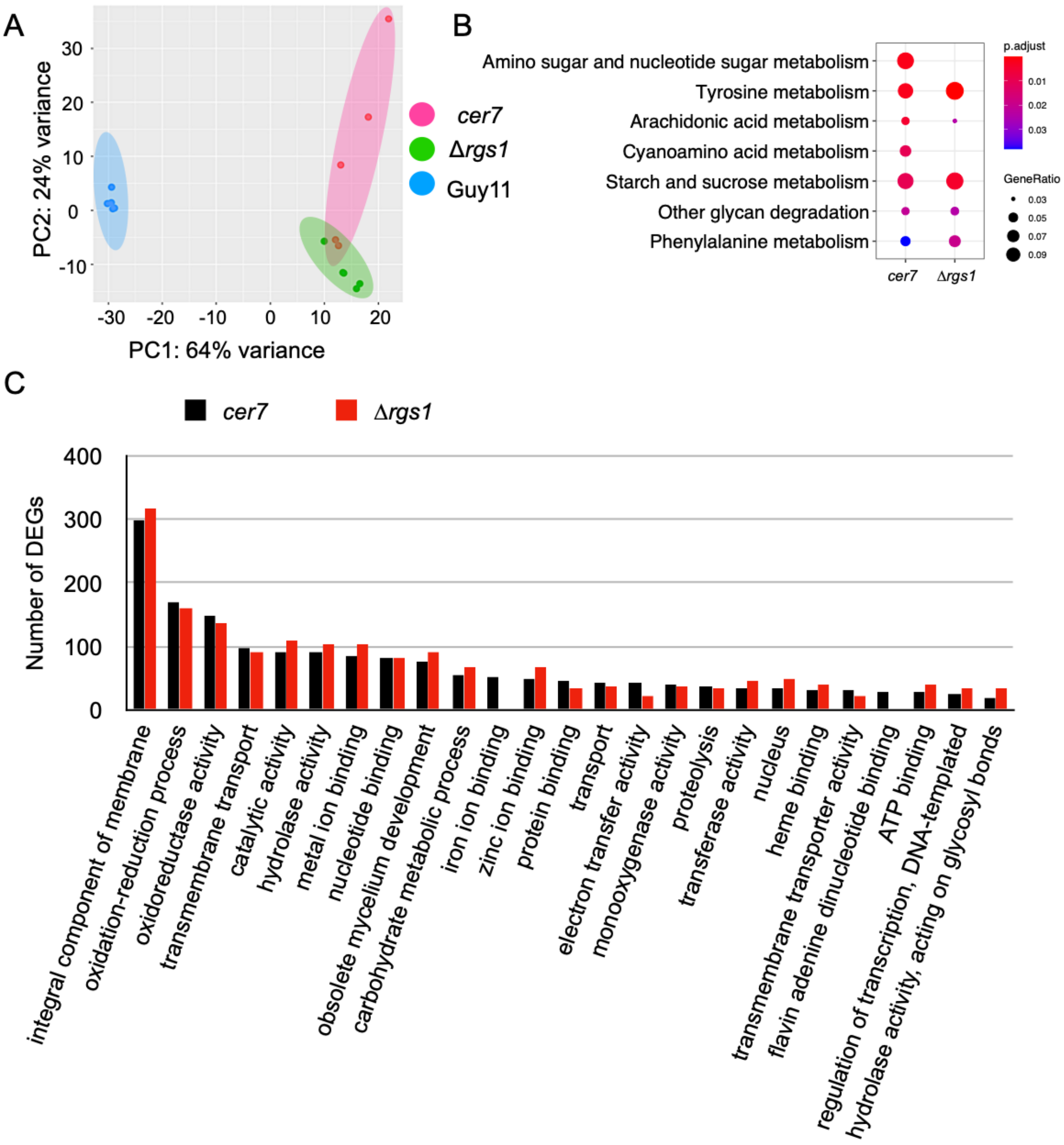

Fig. S6. RNA-seq analysis of *cer7*, *Δrgs1* and Guy11. (***A***) Plot showing Principal Component Analysis (PCA) to determine the distance between samples analysed by RNA-seq. Genes with raw reads counts of less than 10 were discarded, and remaining read counts normalized as FPKMs and used to perform analysis to generate Principal Components (PCs). The x-axis shows PC1, and the y-axis show PC2. Red dots represent samples from the *cer7* mutant, blue dots represent Guy11, and green dots represent the Δ*rgs1* mutant. (***B***) Dot plot showing the comparative metabolic pathway enrichment analysis of DEGs identified from RNA-seq of *cer7* and Δ*rgs1*. The size of dots indicates the ratio of the number of DEGs in the pathway / the number of total DEGs. (***C***) Bar chart showing the top 25 biological processes enriched in DEGs in *cer7* and *Δrgs1* mutants compared to Guy11, based on Gene Ontology analysis of RNA-seq data. The X-axis shows corresponding functions according to the Gene Ontology annotation of *M. oryzae*. The Y-axis shows the number of DEGs identified from the RNA-seq dataset. Black bar plots represent the comparison between *cer7* and Guy11. Red bar plots represent comparison between the *Δrgs1* mutant and Guy11.

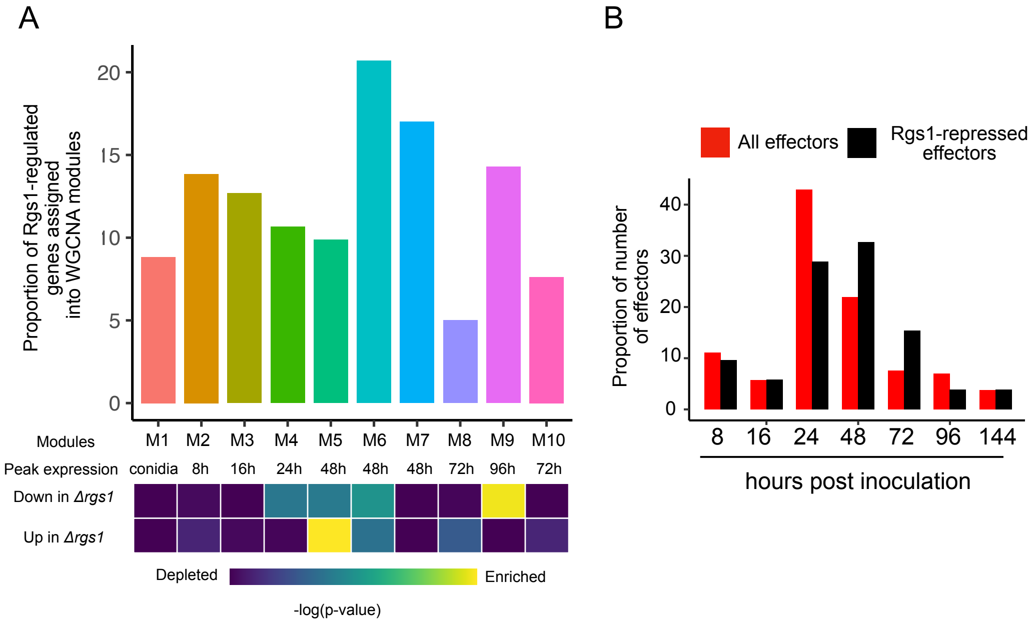

Fig. S7 Rgs1-regulated effectors temporally co-expressed during plant infection. (***A***) Bar chart to show the fraction of Rgs1-regulated genes assigned to each WCGNA co-expression module defined in (1). X-axis shows the time-point of peak expression during plant infection as described previously (1). The heatmap shows enrichment of Rgs1-regulated genes. Enrichment of genes up-regulated in *Δrgs1* conidia was observed in WCGNA modules M4, M5 and M6 which show peak expression at 24-48hpi. Genes down-regulated in *Δrgs1* conidia was observed in M9, which peaks in expression at 96hpi. (***B***). Bar chart to show temporal expression profiles of *M. oryzae* effector-encoding genes (1). Black bars indicate the number of Rgs1-regulated effectors, and red bars the total number of putative effectors expressed at each time point. Rgs1-regulated effectors are over-represented among those peaking in expression at 24h and 48h, consistent with WCGNA co-expression analysis.

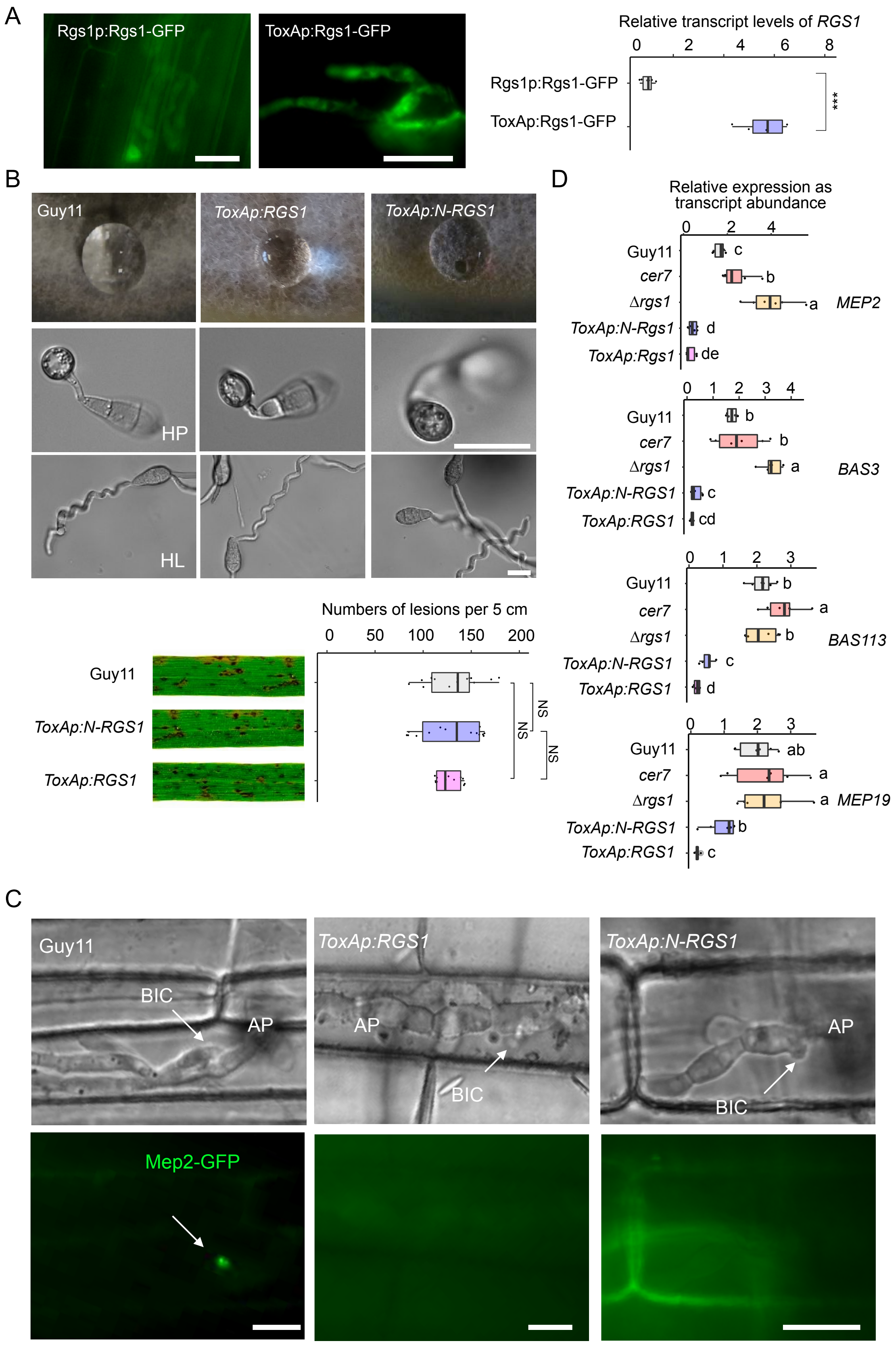
A
Rgs1p:Rgs1-GFP
ToxAp:Rgs1-GFP
Relative transcript levels of RGS1
0 2 4 6 8
Rgs1p:Rgs1-GFP
ToxAp:Rgs1-GFP
***
B
Guy11
ToxAp:RGS1
ToxAp:N-RGS1
HP
HL
Numbers of lesions per 5 cm
0 50 100 150 200
Guy11
ToxAp:N-RGS1
ToxAp:RGS1
NS
NS
NS
D
Relative expression as transcript abundance
0 2 4
Guy11 c
cer7 b
Δrgs1 a
MEP2
ToxAp:N-Rgs1 d
ToxAp:Rgs1 de
0 1 2 3 4
Guy11 b
cer7 b
Δrgs1 a
BAS3
ToxAp:N-RGS1 c
ToxAp:RGS1 cd
0 1 2 3
Guy11 b
cer7 a
Δrgs1 b
BAS113
ToxAp:N-RGS1 c
ToxAp:RGS1 d
0 1 2 3
Guy11 ab
cer7 a
Δrgs1 a
MEP19
ToxAp:N-RGS1 b
ToxAp:RGS1 c
C
Guy11
ToxAp:RGS1
ToxAp:N-RGS1
BIC
AP
Mep2-GFP

365

Fig. S8. Constitutive Rgs1 over-expression prevents Mep2-GFP expression *in planta*. (***A***) Micrographs and boxplot to show expression of Rgs1-GFP driven by the native *RGS1* promoter or over-expression promoter *ToxAp* in invasive hyphae. Rice leaf sheaths of rice cultivar CO39 were inoculated with conidial suspensions and processed for live-cell imaging at 36hpi. (Scale bar, 20 μm). Samples were then collected for total RNA isolation and qRT-PCR to quantify transcripts of *RGS1*. The relative abundance of *RGS1* transcripts as log2 fold change values were calculated against the actin-encoding gene of *M.oryzae*. Significance between groups of samples were performed using Unpaired Student's t-tests. ***p<0.001. (***B***) Micrographs show droplets of water placed on the surface of plate cultures of Guy11, *ToxAp:RGS1*, and *ToxAp:N-RGS1* strains of *M. oryzae* (upper panel). Micrographs show appressorium formation by Guy11, *ToxAp:RGS1*, and *ToxAp:N-RGS1* strains on hydrophobic (HP), and non-inductive hydrophilic surface (HL) (middle panel). (Scale bar, 10μm). Pathogenicity assays were performed using seedlings of rice cultivar CO39 inoculated with conidial suspensions of Guy11, *ToxAp:RGS1*, and *ToxAp:N-RGS1* strains. The boxplot shows the number of rice blast disease lesions per 5 cm of leaf material from two independent repetitions of the experiment. Significance between groups of samples performed using Unpaired Student's t-test. NS = no significant difference. (***C***) Micrographs to show expression and localization of Mep2-GFP at the BIC of invasive hyphae of Guy11, *ToxAp:RGS1*, and *ToxAp:N-RGS1* strains. BIC localization was imaged in rice leaf sheath tissue inoculated with conidial suspensions of Guy11, *ToxAp:RGS1*, and *ToxAp:N-RGS1* strains at 32 hpi. AP (appressoria). (Scale bar, 10 μm). (***D***) Boxplot to show fold changes in relative transcript abundance of genes encoding *MEP2*, *BAS3*, *BAS113*, and *MEP19* in invasive hyphae of *M.oryzae* Guy11, *cer7*, *Δrgs1*, *ToxAp-RGS1*, and *ToxAp-N-RGS1*. Rice leaf sheath were inoculated with conidial suspensions of each strains at 36hpi and used for total RNA isolation and qRT-PCR. Expression was determined relative to the fungal actin encoding gene. Letters refer to significant differences as determined by One-way ANOVA tests (p<0.05, with Duncan's test).

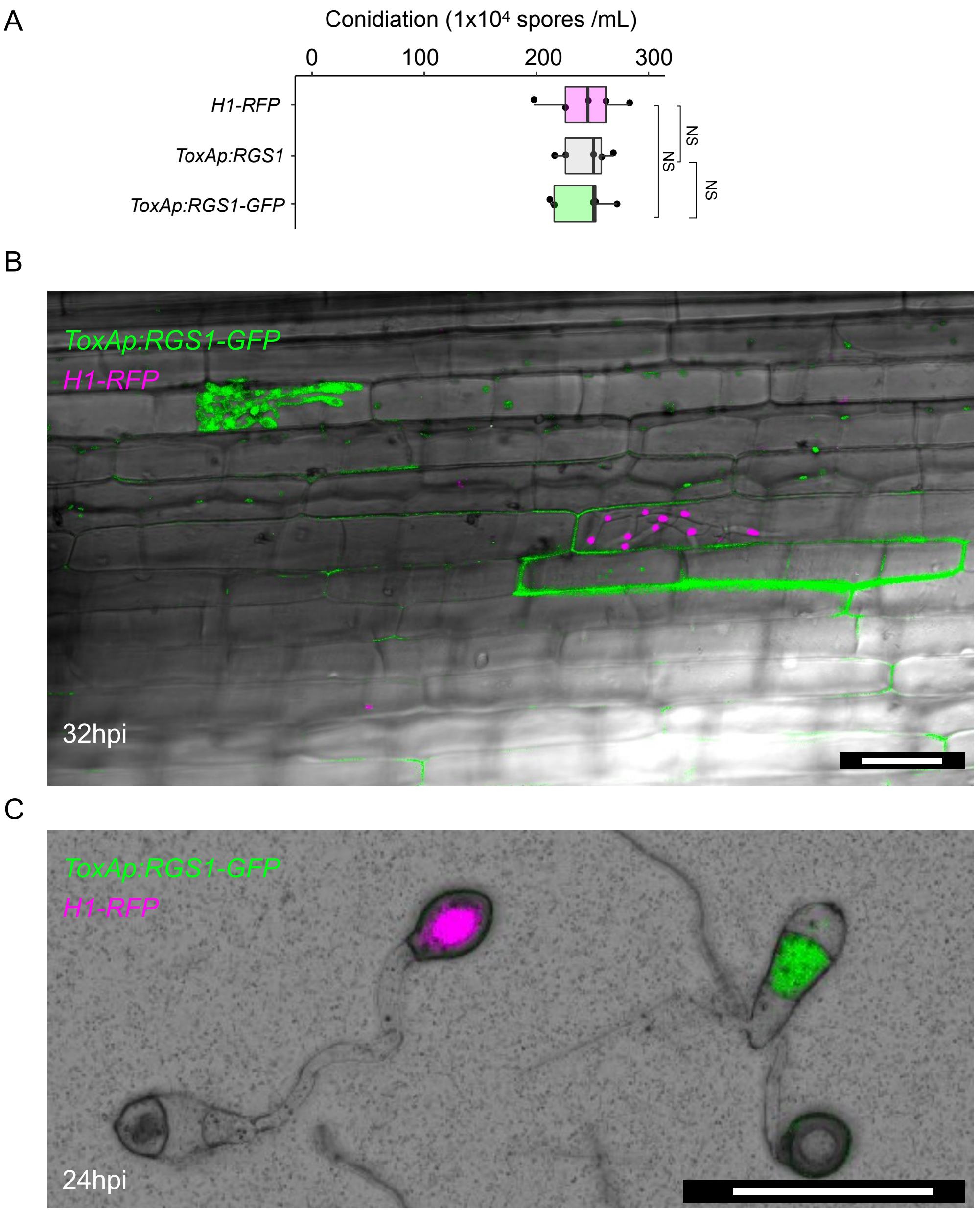

Fig. S9. Relative fitness assay of *M. oryzae ToxAp:RGS1-GFP* and Guy11 *H1-RFP* strains to investigate the consequences of mis-regulation of Rgs1-regulated effectors. (***A***) Boxplot to show the number of conidia produced from axenic cultures of *H1-RFP*, *ToxAp:RGS1*, and *ToxAp:RGS1-GFP* strains. Conidia were harvested and counted from 5-day-old cultures of each strain. Significance between groups of samples was performed using Unpaired Student's t-tests. NS = no significant difference. (***B***) Micrographs to show fluorescence signal of ToxAp:Rgs1-GFP and H1-RFP in invasive hyphae during plant infection. Rice leaf sheath tissue was inoculated with a mixed conidial suspension of ToxAp:Rgs1-GFP and Guy11 H1-RFP and tissue processed for imaging at 32hpi. (Scale bar, 40 μm). (***C***) Micrographs to show the fluorescence signal of ToxAp:Rgs1-GFP and H1-RFP in appressoria that were formed by spores collected from mixture conidial suspension recovered from disease lesions in the relative fitness assay. (Scale bar,15 μm).

Table S1. **Primers used in this study**

Primer name	Sequence (5'-3')	length (bases)
MEP2p-F	CGCGGTGGCGGCCGCTCTAGAGGTACAGGAGGAAGAAGATACA	43
Mep2-R-GFP	GCCCTTGCTCACCATTATGTCAAACCGAACAGGAGGA	37
Rgs1 -F	TGCAGCCCAATGTGGAATTCCTTGGTATGGCCAGAATCGACA	43
Rgs1 -R	TCGACGGTATCGATAAGCTTAGTACGTAACAATCTCGTCTACGA	45
Rgs1-native-allele-P1	CTCGCTCAAGTTCTCCCAGTCAA	24
Rgs1-native-allele-P2	GTCGTGACTGGGAAAACCCTGGCGGTCTGATGAACGAATGACTTGAAG	49
Rgs1-native-allele-P3	TCCTGTGTGAAATTGTTATCCGCTCTACAATATACACCAGGTCTGCCAA	50
Rgs1-native-allele-P4	GGCTTGAGTCAAATGGAATTCG	23
M13F	CGCCAGGGTTTTCCCAGTCACGAC	25
M13R	AGCGGATAACAATTTCACACAGGA	25
Hysplit	GGATGCCTCCGCTCGAAGTA	21
Ygsplit	CGTTGCAAGACCTGCCTGAA	21
Rgs1-KO-1	AGGCGCTATACCCCTTACTGTT	23
Rgs1-KO-2	GTCGTGACTGGGAAAACCCTGGCGAAGCTGAGGGTCTTTCCTTGGA	47
Rgs1-KO-3	TCCTGTGTGAAATTGTTATCCGCTGCATTGTCTTGGTGATGTTTATT	48
Rgs1-KO-4	AATGAGTGACGACCTTGATGT	22
BAS113p-F	TGCAGCCCAATGTGGAATTCAACAATCCCTACCACGTAGCA	42
BAS113-RFP-R	GCCCTTGCTCACCATCCATCCGTTCCACCAGGTGT	36
RFP-F	ATGGTGAGCAAGGGCGA	18
RFP-R	TCGACGGTATCGATAAGCTTCCCTCGAGTGGAGATGTGGAGT	43
Rgs1-GFP-F	CGCGGTGGCGGCCGCTCTAGAGCACACATCATAAAGAAACG	41
Rgs1-GFP-R	TCGACGGTATCGATAAGCTTCGTTGCGAGCGGCTTTGG	38
N-Rgs1-F	TGCAGCCCAATGTGGAATTCGCACACATCATAAAGAAACG	40
N-Rgs1-R	TCGACGGTATCGATAAGCTTGTTGATCATGTCCTTGCCTTTAC	43
C-Rgs1-F	TGCAGCCCAATGTGGAATTCATGGGCTCAGTCCCCAGGG	39
C-Rgs1-R	TCGACGGTATCGATAAGCTTAGTACGTAACAATCTCGTCTACGA	45
RGS1_pGBKT7_F	CATGGAGGCCGAATTCATGGACGACACCTCCCG	34
RGS1_pGBKT7_R	GCAGGTCGACGGATCCTAACCGTTGCGAGCGGCT	35
N-Rgs1-pGBKT7-F	CATGGAGGCCGAATTCATGGACTTTAAGGATTTGTTCGCTAC	43
N-Rgs1-pGBKT7-R	GCAGGTCGACGGATCCTAATTAGTTGATCATGTCCTTGCCTTTAC	45
C-Rgs1-pGBKT7-F	CATGGAGGCCGAATTCATGCCAAATGTCAAGTCTAGCGTCA	42
C-Rgs1-pGBKT7-R	GCAGGTCGACGGATCCTAAGATCATGTCCTTGCCTTTACTTG	43
RGS1_pGBGT7_F	ATGGCCATGGAGGCCAGTATGGACGACACCTCCCG	36
RGS1_pGBGT7_R	AGCTCGAGCTCGATGAACCGTTGCGAGCGGCT	33
N-Rgs1-pGBGT7-F	ATGGCCATGGAGGCCAGTATGGACTTTAAGGATTTGTTCGCTAC	45
N-Rgs1-pGBGT7-R	AGCTCGAGCTCGATGTAATTAGTTGATCATGTCCTTGCCTTTAC	44
C-Rgs1-pGBGT7-F	ATGGCCATGGAGGCCAGTATGCCAAATGTCAAGTCTAGCGTCA	44

C-Rgs1-pGBGT7-R	AGCTCGAGCTCGATGTAAGATCATGTCCTTGCCTTTACTTG	42
MEP2-promoter-F	AAATGATGAATTGAAAAGCTTTCCTCCTGTTCGGTTTGACA	41
MEP2-promoter-R	ATACAGAGCACATGCCTCGAGCATTTAGCTGAGCTTGGTC	40
Rgs1-cDNA-F	GCCATGGAGGCCAGTGAATTCATGGACGACACCTCCCG	38
Rgs1-cDNA-R	ACGATTCATCTGCAGCTCGAGTAACCGTTGCGAGCGGCT	39
MagA-G187S-p1	TGCAGCCCAATGTGGAATTCATCTCGACGAATACCATGCGT	41
MagA-G187S-p2	GCTTGTAGTTTTTGTTCTTGC	21
MagA-G187S-p3	ACAAAAACTACAAGCATATACGA	23
MagA-G187S-p4	TCGACGGTATCGATAAGCTTTCGTCGTGCGATCTCTGC	38
MagA-Q208L-p1	TGCAGCCCAATGTGGAATTCATCTCGACGAATACCATGCGT	41
MagA-Q208L-p2	CCTGCTGCCTCCTACATCAAA	21
MagA-Q208L-p3	GTAGGAGGCAGCAGGAGTGAGC	22
MagA-Q208L-p4	TCGACGGTATCGATAAGCTTTCGTCGTGCGATCTCTGC	38
MagB-G183S-p1	TGCAGCCCAATGTGGAATTCCATCTATCAAAGCAAGTCGCCA	42
MagB-G183S-p2	CTCAGTGATACTGGTTGTCTTG	22
MagB-G183S-p3	ACCAGTATCACTGAGACGACTT	22
MagB-G183S-p4	TCGACGGTATCGATAAGCTTTGCCTGGTGTAATTCGGCT	39
MagB-Q204L-p1	TGCAGCCCAATGTGGAATTCCATCTATCAAAGCAAGTCGCCA	42
MagB-Q204L-p2	GAACGGCTGCCACCAACATC	20
MagB-Q204L-p3	TGGTGGCAGCCGTTCTGAGC	20
MagB-Q204L-p4	TCGACGGTATCGATAAGCTTTGCCTGGTGTAATTCGGCT	39
MagB-G42R-p1	TGCAGCCCAATGTGGAATTCCATCTATCAAAGCAAGTCGCCA	42
MagB-G42R-p2	TTCACGGGCTCCGAGCAGAA	20
MagB-G42R-p3	CTCGGAGCCCGTGAATCCG	19
MagB-G42R-p4	TCGACGGTATCGATAAGCTTTGCCTGGTGTAATTCGGCT	39
MagC-G184s-p1	TGCAGCCCAATGTGGAATTCTACCCACCTTGTCCACCTT	39
MagC-G184s-p2	GCACATCTTGCCAGAGaGCAATTGGCGGATTGCTCC	36
MagC-G184s-p3	GGAGCAATCCGCCAATTGCCCTCTGGCAAGATGTGC	36
MagC-G184s-p4	TCGACGGTATCGATAAGCTTAGCCTCCACTGGAACTAGCCC	41
BAS3p-F	TGCAGCCCAATGTGGAATTCCATCTGCAGCGACGATCTGT	40
BAS3-RFP-R	GCCCTTGCTCACCATCTGGGCACTGTTGGCAGCG	34
N-Rgs1_p1	TGCAGCCCAATGTGGAATTC GCACACATCATAAAGAAACG	41
N-Rgs1_p2	GTTGATCATGTCCTTGCCTT	20
N-Rgs1_p3	AAGGACATGATCAACTGAAATTAATCCAGCC	31
N-Rgs1_p4	TCGACGGTATCGATAAGCTTGGCTTGAGTCAAATGGAATTCG	42
C-Rgs1_p1	TGCAGCCCAATGTGGAATTCGCACACATCATAAAGAAACG	40
C-Rgs1_p2	CTACCACCGAACAAAAGCTGAG	22
C-Rgs1_p3	TTTTGTTCGGTGGTAGATGGGCTCAGTCCCCAGG	34
C-Rgs1_p4	GACGGTATCGATAAGCTTGGCTTGAGTCAAATGGAATTCG	40
MEP2-F	TTAGCCCTTGCTGCTGC	17

MEP2-R	TTGGCTTCACCAAACG	16
Mo-actin-F	GTGACTTGACCGACTACCTGA	22
Mo-actin-R	TGCCGATGGTGATAACCTGT	21
β-tubulin-F	CTGCCATCTTCCGTGGAAAGG	22
β-tubulin-R	GACGAAGTACGACGAGTTCTTG	23
PR1a-F	TGGGTGTCGGAGAAGCAGTG	24
PR1a-R	GGTGATGAAGACGCCGAGG	20
PR10b-F	ATGTTACTGTGGCGTGGTCGCATGT	31
PR10b-R	TGTGTATTTTATTCACTCGTGAAGCA	27
CPS2-F	CAATCGAGATCTGCTCAGGA	26
CPS2-R	CTGGCGTAGTCTGCACTGTT	21
Rice-AK061464-F	TTTCACTCTTGGTGTGAAGCAGAT	24
Rice-AK061464-R	GACTTCCTTCACGATTTCATCGTAA	25
Rice-AK061988-F	ACCACTTCGACCGCCACTACT	21
Rice-AK061988-R	ACGCCTAAGCCTGCTGGTT	19
ToxA_N-Rgs1_F	TGCAGCCCAATGTGGAATTCCGATTGGAATGCATGGAGGAG	41
ToxA_N-Rgs1_R	GGACTATATTCATTCAATGTCAGCGGGAGGTGTCGTCCAT	40
MagA_L_1	ATCTCGACGAATACCATGC	19
MagA_L_2	GTCGTGACTGGGAAAACCCTGGCGATCATGAATGGTCCTGTTGG	45
MagA_L_1nest	TACGTTGAGTGCAATCATTGG	22
MagA_R_1	TGATGGTTCAGTTCCACAGG	21
MagA_R_2	TCCTGTGTGAAATTGTTATCCGCTTCATTCTAGTTCCTTCCTTATAC	48
MagA_R_1nest	TGAGATGCCATGACTTGGCA	21
MagB_L_1	CATCTATCAAAGCAAGTCGCCA	23
MagB_L_2	GTCGTGACTGGGAAAACCCTGGCGGAACGATAATTAATACAAGCGATG	49
MagB_L_1nest	GGCAGCTTAATATGGGGGTAT	22
MagB_R_1	GAGCAGCAAATGTACAACTGAA	23
MagB_R_2	TCCTGTGTGAAATTGTTATCCGCTCTAGAGCACCATCACTACCA	45
MagB_R_1nest	CATCGTGGTTTGATGGCATTTC	23
MagC_L_1	ACATACCTAGTGTAGGTACCTAG	24
MagC_L_2	GTCGTGACTGGGAAAACCCTGGCGGAAGCACATCTTGCCAGAG	44
MagC_L_1nest	GTTACCATGCCAGGCTACTGA	22
MagC_R_1	GAACTAGCCCCATAACTATGTCG	24
MagC_R_2	TCCTGTGTGAAATTGTTATCCGCTGGAAATTCTGCCACATCG	43
MagC_R_1nest	AACTATGTCGTTGTTATGAGCCA	24
qPCRbas113_F	AACAGCTTGTTCACAC	17
qPCRbas113_R	TGGACTGACGTCGGGG	17
qPCRbas3_F	TCCACCGTCTCCTTT	16
qPCRbas3_R	AGCGCTGCCTTCAGAT	17
qPCRMep2_F	TTAGCCCTTGCTGCTGC	17

qPCRMep2_R	TTGGCTTCACCAAACG	16
qPCRNrgs1_F	CCCATCCGAGGTCGTC	17
qPCRNrgs1_R	AGCCCCCGACTCCAAG	17
qPCRMep19_F	TGGTTCTCCCCATCC	15
qPCRMep19_R	GAATGCAACAGTAT	14

Table S2 *Magnaporthe oryzae* strains generated in this study

Strain Name	Description
cer7	Guy11 constitutively expressing Mep2-GFP after UV mutagenesis
Mep2-GFP	Guy11 transformed with plasmid pMep2-GFP
cer7/Rgs1cer7	cer7 transformed with plasmid Rgs1cer7
cer7/Rgs1wt	cer7 transformed with plasmid RGs1wt
Δrgs1	targeted gene deletion in Rgs1 locus
Rgs1CER7	Guy11 transformed with plasmid pMep2-GFP and transformed with native allele of Rgs1 carrying the Cer7 mutant
Δrgs1/Mep2-GFP	Δrgs1 transformed with plasmid pMep2-GFP
Mep2-GFP/rgs1cer7	Guy11 strain with Mep2-GFP and transformed with plasmid rgs1cer7
pToxA-GFP	Guy11 with constitutively expressed GFP driven by pToxA
Rgs1-GFP	Guy11 transformed with plasmid Rgs1-GFP
cer7/N-Rgs1	cer7 transformed with plasmid N-Rgs1
cer7/C-Rgs1	cer7 transformed with plasmid C-Rgs1
MagA-Q208L	Guy11 constitutively expressing Mep2-GFP and transformed with plasmid MagA-Q208L
MagA-G187S	Guy11 constitutively expressing Mep2-GFP and transformed with plasmid MagA-G187S
MagB-G183S	Guy11 constitutively expressing Mep2-GFP and transformed with plasmid MagB-G183S
MagB-Q204L	Guy11 constitutively expressing Mep2-GFP and transformed with plasmid MagB-Q204L
MagB-G42R	Guy11 constitutively expressing Mep2-GFP and transformed with plasmid MagA-Q208L
MagC-G184S	Guy11 constitutively expressing Mep2-GFP and transformed with plasmid MagC-G184S
cer7/bas113-RFP	cer7 transformed with plasmid Bas113-RFP
cer7/Bas3-RFP	Ccer7 transformed with plasmid Bas3-RFP
ToxAp-Rgs1-GFP	Guy11 transformed with plasmid ToxAp-Rgs1-GFP
ToxAp-N-rgs1	Guy11 transformed with plasmid ToxAp-N-Rgs1
ΔmagA/cer7	targeted gene deletion in MagA locus using cer7 strain
ΔmagB/cer7	targeted gene deletion in MagB locus using cer7 strain
ΔmagC/cer7	targeted gene deletion in MagC locus using cer7 strain

Table S3 Rgs1-regulated effectors identified in this study

Gene Locus Number	Gene Name	Reference
MGG_07986	*CDIP3*	Chen, Songbiao, Pattavipha Songkumarn, R. C. Venu, Malali Gowda, Maria Bellizzi, Jinnan Hu, Wende Liu et al. "Identification and characterization of in planta–expressed secreted effector proteins from *Magnaporthe oryzae* that induce cell death in rice." *Molecular plant-microbe interactions* 26, no. 2 (2013): 191-202.
MGG_10532	*NLP4*	Fang, Ya-Li, You-Liang Peng, and Jun Fan. "The Nep1-like protein family of *Magnaporthe oryzae* is dispensable for the infection of rice plants." *Scientific reports* 7, no. 1 (2017): 1-10.
MGG_05785	*BAS113*	Giraldo, Martha C., Yasin F. Dagdas, Yogesh K. Gupta, Thomas A. Mentlak, Mihwa Yi, Ana Lilia Martinez-Rocha, Hiromasa Saitoh, Ryohei Terauchi, Nicholas J. Talbot, and Barbara Valent. "Two distinct secretion systems facilitate tissue invasion by the rice blast fungus *Magnaporthe oryzae*." *Nature communications* 4, no. 1 (2013): 1-12.
MGG_11610	*BAS3*	Mosquera, Gloria, Martha C. Giraldo, Chang Hyun Khang, Sean Coughlan, and Barbara Valent. "Interaction transcriptome analysis identifies *Magnaporthe oryzae* BAS1-4 as biotrophy-associated secreted proteins in rice blast disease." *The Plant Cell* 21, no. 4 (2009): 1273-1290.
MGG_02154	*SPD5*	Sharpee, William, Yeonyee Oh, Mihwa Yi, William Franck, Alex Eyre, Laura H. Okagaki, Barbara Valent, and Ralph A. Dean. "Identification and characterization of suppressors of plant cell death (SPD) effectors from *Magnaporthe oryzae*." *Molecular plant pathology* 18, no. 6 (2017): 850-863.
MGG_00230	*MEP2*	Yan, Xia, Bozeng Tang, Lauren Ryder, Dan Maclean, Vincent M. Were, Alice Bisola Eseola, Neftaly Cruz-Mireles, Andy Foster, Miriam Osés-Ruiz, and Nicholas J. Talbot. "The transcriptional landscape of plant infection by the rice blast fungus *Magnaporthe oryzae* reveals distinct families of temporally co-regulated and structurally conserved effectors." *bioRxiv* (2022).
MGG_15443	*MEP27*	Yan, Xia, Bozeng Tang, Lauren Ryder, Dan Maclean, Vincent M. Were, Alice Bisola Eseola, Neftaly Cruz-Mireles, Andy Foster, Miriam Osés-Ruiz, and Nicholas J. Talbot. "The transcriptional landscape of plant infection by the rice blast fungus *Magnaporthe oryzae* reveals distinct families of temporally co-regulated and structurally conserved effectors." *bioRxiv* (2022).
MGG_10004	*MEP19*	Yan, Xia, Bozeng Tang, Lauren Ryder, Dan Maclean, Vincent M. Were, Alice Bisola Eseola, Neftaly Cruz-Mireles, Andy Foster, Miriam Osés-Ruiz, and Nicholas J. Talbot. "The transcriptional landscape of plant infection by the rice blast fungus *Magnaporthe oryzae* reveals distinct families of temporally co-regulated and structurally conserved effectors." *bioRxiv* (2022).
MGG_14053		
MGG_16703		
MGG_10318		
MGG_10074		
MGG_07411		
MGG_08957		
MGG_08372		
MGG_12551		
MGG_16175		
MGG_00732		
MGG_08373		
MGG_16026		

MGG_00081
MGG_18035
MGG_16989
MGG_08789
MGG_05424
MGG_16238
MGG_09848
MGG_06234
MGG_04737
MGG_17666
MGG_16869
MGG_14006
MGG_16714
MGG_03806
MGG_03585
MGG_02234
MGG_08941
MGG_08817
MGG_17266
MGG_05638
MGG_14603
MGG_00052
MGG_05406
MGG_09709
MGG_17522
MGG_17894
MGG_02223
MGG_15689
MGG_05108
MGG_16339
MGG_01963
MGG_16422
MGG_16382
MGG_07993
MGG_09128
MGG_01173
MGG_11991
MGG_04973
MGG_00992
MGG_04889

SI References

1. X. Yan *et al.*, The transcriptional landscape of plant infection by the rice blast fungus Magnaporthe oryzae reveals distinct families of temporally co-regulated and structurally conserved effectors. *bioRxiv* (2022).
2. H. Liu *et al.*, Rgs1 regulates multiple Gα subunits in Magnaporthe pathogenesis, asexual growth and thigmotropism. *The EMBO journal* **26**, 690-700 (2007).
3. R. J. Lindsay, M. J. Kershaw, B. J. Pawlowska, N. J. Talbot, I. Gudelj, Harbouring public good mutants within a pathogen population can increase both fitness and virulence. *eLife* **5**, e18678 (2016).
4. L. S. Ryder *et al.*, A sensor kinase controls turgor-driven plant infection by the rice blast fungus. *Nature* **574**, 423-427 (2019).
5. N. J. Talbot, D. J. Ebbole, J. E. Hamer, Identification and characterization of MPG1, a gene involved in pathogenicity from the rice blast fungus Magnaporthe grisea. *The Plant Cell* **5**, 1575-1590 (1993).
6. M. J. Kershaw, N. J. Talbot, Genome-wide functional analysis reveals that infection-associated fungal autophagy is necessary for rice blast disease. *Proceedings of the National Academy of Sciences* **106**, 15967 (2009).
7. W. Sakulkoo *et al.*, A single fungal MAP kinase controls plant cell-to-cell invasion by the rice blast fungus. *Science* **359**, 1399 (2018).
8. N. L. Catlett, B.-N. Lee, O. Yoder, B. G. Turgeon, Split-marker recombination for efficient targeted deletion of fungal genes. *Fungal Genetics Newsletter*, 9-11 (2003).
9. M. J. Kershaw *et al.*, Conidial morphogenesis and septin-mediated plant infection require Smo1, a Ras GTPase-activating protein in Magnaporthe oryzae. *Genetics* **211**, 151-167 (2019).
10. N. J. Talbot *et al.*, MPG1 encodes a fungal hydrophobin involved in surface interactions during infection-related development of Magnaporthe grisea. *The Plant Cell* **8**, 985-999 (1996).
11. A. M. Bolger, M. Lohse, B. Usadel, Trimmomatic: a flexible trimmer for Illumina sequence data. *Bioinformatics* **30**, 2114-2120 (2014).
12. B. Langmead, S. L. Salzberg, Fast gapped-read alignment with Bowtie 2. *Nat Methods* **9**, 357-359 (2012).
13. D. C. Koboldt *et al.*, VarScan 2: somatic mutation and copy number alteration discovery in cancer by exome sequencing. *Genome Res* **22**, 568-576 (2012).
14. J. T. Robinson *et al.*, Integrative genomics viewer. *Nature biotechnology* **29**, 24-26 (2011).
15. Y. Liao, G. K. Smyth, W. Shi, The R package Rsubread is easier, faster, cheaper and better for alignment and quantification of RNA sequencing reads. *Nucleic acids research* **47**, e47-e47 (2019).
16. G. S. C. Slater, E. Birney, Automated generation of heuristics for biological sequence comparison. *BMC bioinformatics* **6**, 1-11 (2005).
17. J. T. Leek, W. E. Johnson, H. S. Parker, A. E. Jaffe, J. D. Storey, The sva package for removing batch effects and other unwanted variation in high-throughput experiments. *Bioinformatics* **28**, 882-883 (2012).
18. G. Yu, L.-G. Wang, Y. Han, Q.-Y. He, clusterProfiler: an R package for comparing biological themes among gene clusters. *OMICS* **16**, 284-287 (2012).
19. K. J. Livak, T. D. Schmittgen, Analysis of Relative Gene Expression Data Using Real-Time Quantitative PCR and the 2−ΔΔCT Method. *Methods* **25**, 402-408 (2001).
20. P. Kankanala, K. Czymmek, B. Valent, Roles for rice membrane dynamics and plasmodesmata during biotrophic invasion by the blast fungus. *The Plant Cell* **19**, 706-724 (2007).
21. J. N. Abelson, M. I. Simon, C. Guthrie, G. R. Fink, *Guide to yeast genetics and molecular biology* (Gulf Professional Publishing, 2004).
